# Amphiregulin contained in NSCLC-exosomes induces osteoclast differentiation through the activation of EGFR pathway

**DOI:** 10.1038/s41598-017-03460-y

**Published:** 2017-06-09

**Authors:** Simona Taverna, Marzia Pucci, Marco Giallombardo, Maria Antonietta Di Bella, Mariacarmela Santarpia, Pablo Reclusa, Ignacio Gil-Bazo, Christian Rolfo, Riccardo Alessandro

**Affiliations:** 1 Biopathology and Biomedical Methodology, Biology and Genetic section, University of Palermo, Palermo, Italy; 2 Institute of Biomedicine and Molecular Immunology (IBIM), National Research Council, Palermo, Italy; 3 Medical Oncology Unit, Department of Human Pathology “G. Barresi”, University of Messina, Messina, Italy; 4 Phase I-Early Clinical Trials Unit, Oncology Department, Antwerp University Hospital (UZA) and Center for Oncological Research (CORE) Antwerp University, Antwerp, Belgium; 5 Clinica Universidad de Navarra – Center for Applied Medical Research, Pamplona, Spain

## Abstract

Non-small cell lung cancer (NSCLC) remains the leading cause of cancer-related deaths worldwide. The majority of patients are diagnosed in advanced disease stage. Bone metastasis is the most frequent complication in NSCLC resulting in osteolytic lesions. The perfect balance between bone-resorbing osteoclasts and bone-forming osteoblasts activity is lost in bone metastasis, inducing osteoclastogenesis. In NSCLC, the epidermal growth factor receptor (EGFR) pathway is constitutively activated. EGFR binds Amphiregulin (AREG) that is overexpressed in several cancers such as colon, breast and lung. Its levels in plasma of NSCLC patients correlate with poor prognosis and AREG was recently found as a signaling molecule in exosomes derived from cancer cell lines. Exosomes have a key role in the cell-cell communication and they were recently indicated as important actors in metastatic niche preparation. In the present work, we hypothesize a role of AREG carried by exosomes derived from NSCLC in bone metastasis induction. We observed that NSCLC-exosomes, containing AREG, induce EGFR pathway activation in pre-osteoclasts that in turn causes an increased expression of RANKL. RANKL is able to induce the expression of proteolytic enzymes, well-known markers of osteoclastogenesis, triggering a vicious cycle in osteolytic bone metastasis.

## Introduction

Non-small cell lung cancer is one of the most commonly diagnosed neoplasia and the main cause of cancer-related deaths in Asian and Western populations^1^. Surgery and chemotherapy are the two major treatments to prolong the survival of NSCLC patients^2^. About 50% of lung cancer cases are metastatic at diagnosis, with preferential localization in bone, brain, liver and distant lymph node^3^. 19–33% of NSCLC-patients show bone metastasis at diagnosis, while 41% develop bone metastasis during the course of disease^4^. Lung cancer induces the formation of both osteoblastic and osteolytic metastases and, in NSCLC lytic bone metastasis are the most common^5, 6^.

In NSCLC the epidermal growth factor receptor (EGFR) signaling is up regulated^7^. EGFR phosphorylation leads to the activation of downstream signalling inducing different proto-oncogenes. EGFR binds several ligands among which Amphiregulin (AREG) attracted our attention for several reasons. AREG is overexpressed in several cancers such as colon, breast and lung^8^. Moreover, its levels in plasma of NSCLC patients correlate with poor prognosis^9^ and was recently found as a signaling molecule in exosomes derived from cancer cell lines^10^.

Several studies demonstrated that exosomes have a key role in the cell-cell communication^11–14^ and they recently were indicated as important actors in metastatic niche preparation^15, 16^. Higginbotham and colleagues showed that exosomes released by cancer cells contain high level of AREG^10^. EGFR ligand signaling via exosomes might contribute to cancer progression in order to prepare the metastatic niche^17^.

Recently, it was demonstrated that EGFR is expressed in pre-osteoclasts and EGFR signaling is necessary for osteoclast formation from bone marrow precursor cell^8, 18^.

NSCLC cells induce the release of factors that alter bone remodeling increasing osteoclast activity through the shift of the normal balance between Receptor-Activator-of-Nuclear-factor-Kappa-B-Ligand (RANKL) and Osteoprotegerin (OPG)^18^. This bone destructive process induces a “vicious cycle” in which growth factors released by the osteoclasts are able to stimulate tumor growth and molecules released by cancer cells in turn enhance the osteoclast differentiation. EGFR can modulate RANKL-activated signaling pathways through a cross talk with RANK/RANKL system. EGFR ligands stimulate osteoclast formation by inhibiting the OPG expression and upregulating RANKL^19^.

EGFR is the target of epidermal growth factor receptor–tyrosine kinase inhibitors, EGFR–TKIs (such as Gefitinib and Erlotinib). EGFR–TKIs show a strong antitumor activity in a subset of NSCLC patients, with activating mutations in EGFR gene. Among EGFR-TKI, Erlotinib binds to EGFR intracellular tyrosine kinase domain, blocks autophosphorylation of EGFR with subsequent inhibition of the downstream signaling cascades^17, 20^. Erlotinib is used as standard treatment for previously treated advanced NSCLC.

Our research group demonstrated that exosomes released by multiple myeloma cells are involved in osteoclasts differentiation. These exosomes induced the differentiation of murine RAW 264.7 and human primary preosteoclasts in osteoclasts, increasing the expression of osteoclast markers such as Cathepsin K (CTSK), Matrix Metalloproteinases 9 (MMP9) and Tartrate-resistant Acid Phosphatase (TRAP)^20^.

Now, we hypothesized a role of AREG carried by exosomes derived from NSCLC in bone metastasis induction. We observed that NSCLC-exosomes, containing AREG, induced EGFR pathway activation that in turn caused osteoclasts differentiation. AREG knockdown, neutralizing antibodies for AREG and the co-treatment with NSCLC-exosomes and Erlotinib reverted the osteoclast differentiation induced by exosomes.

## Results

### Characterization of exosomes released by NSCLC cells

**Figure 1:**
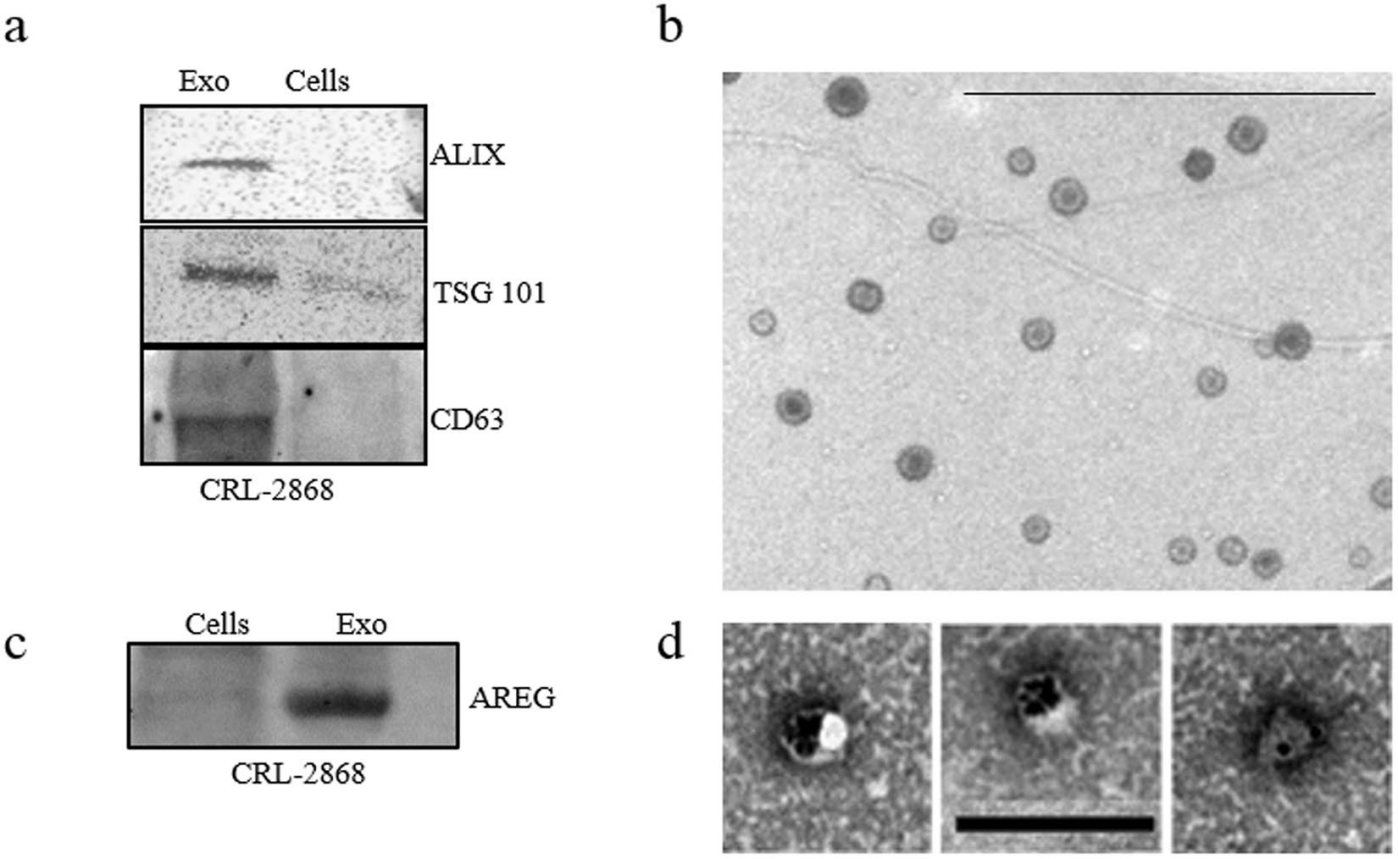
CRL-2868 Exosomes characterization (**a**) Detection by western blotting of ALIX, TSG 101 and CD63 in 30 µg of exosomes purified conditioned medium of CRL-2868 cells compared to 30 µg of parental cells whole lysate. Original uncropped WBs were reported in Figure S5A. (**b**) Representative negative EM image of exosomes released by CRL-2868 cells. Scale bar 500 nm. (**c**) Detection by western blotting of AREG in 30 µg of CRL-2868 exosomes compared to 30 µg of parental cells whole lysate. Original uncropped WBs were reported in Figure S5B. (**d**) Gold labelling immune-electron microscopy and negative staining indicating the presence of AREG antigen on exosomes. Scale bar 200 nm.

Vesicles released by CRL-2868 cells into the culture medium were isolated, purified on a sucrose gradient and characterized as exosomes as previously described^13^. Vesicles were analyzed by Western blotting using antibodies specific for ALIX, TSG 101 and CD63 to confirm their exosomal identity (Fig. 1a). TEM analysis further showed vesicles with well-known cup shape already described for exosomes isolated from different cell lines (Fig. 1b).

### NSCLC-exosomes contain Amphiregulin

In NSCLC cells, EGFR signaling is constitutively activated^7^. EGFR is bound and activated by a family of seven peptide growth factors among which we focused on Amphiregulin; we observed that CRL-2868 exosomes were enriched in AREG with respect to parental cells (Fig. 1c). TEM analyses following immunogold-labelling show that AREG decorates the exosomal membranes (Fig. 1d). Moreover, we demonstrated that exosomes^21–23^ released by other three cell lines (NSCLC: A549; Prostate cancer: PC3; Breast Cancer: MDA-MB 231) inducing bone metastases, are enriched in AREG compared to parental cells (Figure S1a).

### Raw 264.7 cells internalize CRL-2868 exosomes

**Figure 2:**
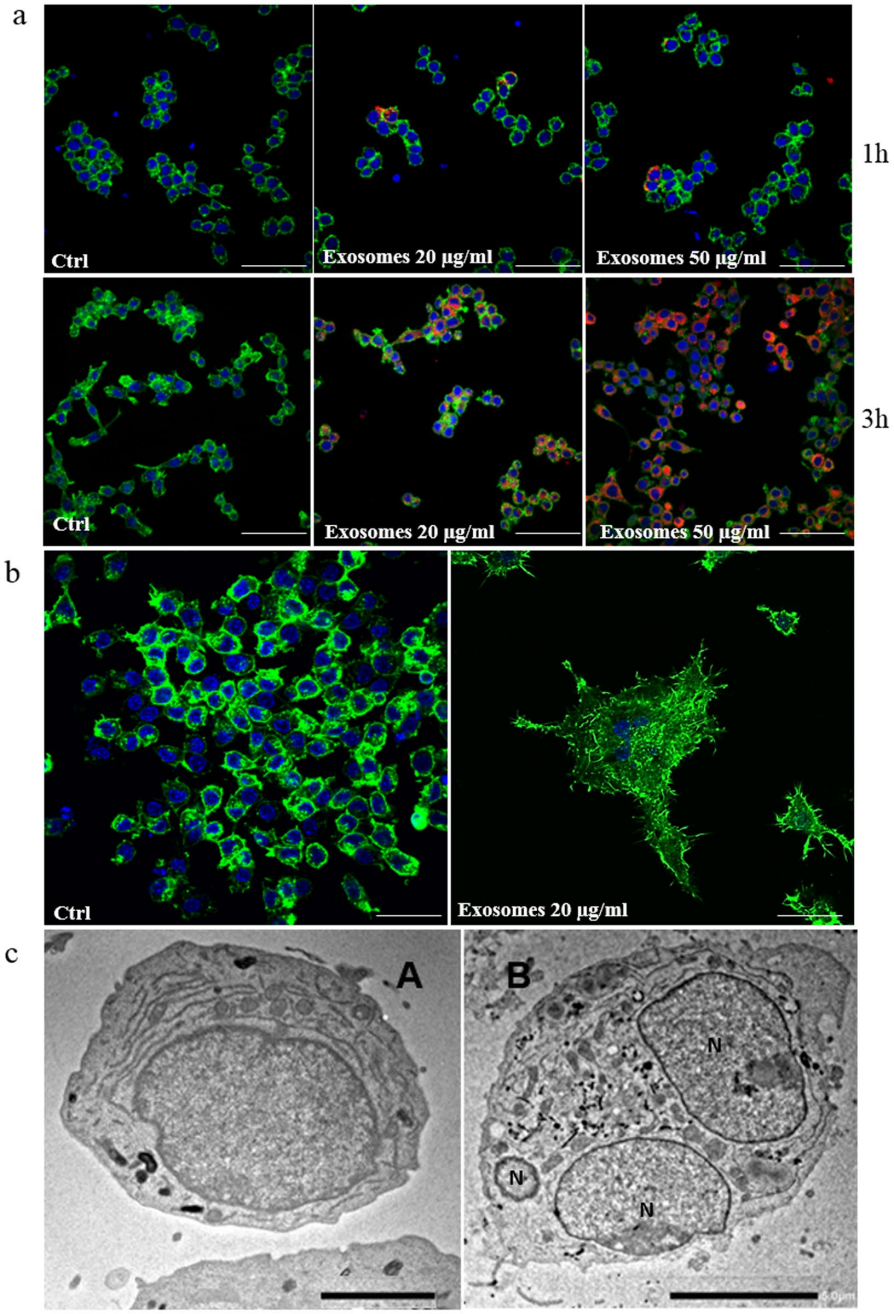
CRL-2868 exosomes are internalized by RAW 264.7 cells and induce preosteoclasts morphological differentiation. (**a**) Confocal microscopy analysis of RAW 264.7 cells treated, for 1 and 3 hours, with 20 μg/ml (Exosomes 20 μg/ml) and 50 μg/ml (Exosomes 50 μg/ml) of CRL-2868 exosomes, compared to untreated RAW 264.7 cells (Ctrl). RAW 264.7 were stained with ActinGreen (green), nuclear counterstaining was performed using Hoescht (blue); exosomes were labelled with PKH26 (red). Scale bar 50 µm. (**b**) Confocal microscopy analysis of RAW 264.7 cells treated, for 6 days with 20 μg/ml (Exosomes 20 μg/ml) of CRL-2868 exosomes, compared to untreated RAW 264.7 cells (Ctrl). Scale bar 10 µm. (**c**) Comparative morphological features of pre-osteoclasts grown in the absence (A) and presence (B) of CRL-2868 exosomes and analyzed by TEM. Scale bars: A = 2 µm; B = 5 µm.

The ability of CRL-2868 exosomes, to be transferred to RAW 264.7 cells, was investigated by examining the uptake of isolated exosomes labeled with PKH-26. RAW 264.7 cells treated with CRL-2868 exosomes internalized the vesicles in a time and dose-dependent manner (Fig. 2a). We also observed that exosomes released by A549, NSCLC cell line, are uptaken by RAW 264.7 cells in time and dose dependent manner (Figure S1b). However, the uptake of CRL-2868 and A549 exosomes in RAW 264.7 cells was blocked by treatment of pre-osteoclasts with 50 μM EIPA (Figure S1c), a known blocker of macropinocytosis thus confirming that exosomes internalization was mediated by endocitosis as previously described^24^. Semi-quantitative analysis of PKH-26-exosomes fluorescence intensity in the cytoplasm of RAW 264.7 cells is showed in Figure S2a and b.

### CRL-2868 exosomes induce morphological differentiation of preosteoclasts

The addition of CRL-2868 exosomes to RAW 264.7 cells positively modulates cell differentiation in mature osteoclasts inducing the typical osteoclast morphology. In Fig. 2b confocal analyses of RAW 264.7 treated with CRL-2868 exosomes showed the induction of multinucleated cells and filopodia formation. Exosomes released by A549 cells, are able to induce osteoclast differentiation similarly to exosomes released by CRL-2868 cells (Figure S2c), inducing multinucleated morphology of pre-osteoclasts, typical of mature osteoclasts.

Furthermore, in order to evaluate the differentiation toward a mature osteoclast phenotype we analyzed the ultrastructural morphology of cells at TEM. In the control culture (untreated RAW 264.7 cells), cells displayed the typical features of monocytes showing smooth cell surfaces (Fig. 2c, (A). On the contrary, multinucleated cells of various size and configuration were observed among monocytes grown in the presence of CRL-2868 exosomes (Fig. 2c, (B). The multinucleated osteoclasts were structurally characterized by the development of ruffled borders; they exhibited large nuclei with several nucleoli. In the cytoplasm, we observed a rich rough endoplasmic reticulum, mitochondria and many lysosomal bodies.

### CRL-2868 exosomes induce the activation of EGFR pathway

It was demonstrated that EGFR is expressed in pre-osteoclasts and EGFR signaling is necessary for osteoclast formation from bone marrow precursor cells. EGFR ligands stimulate osteoclast formation by inhibiting OPG expression and upregulating msRANKL^17^.

**Figure 3:**
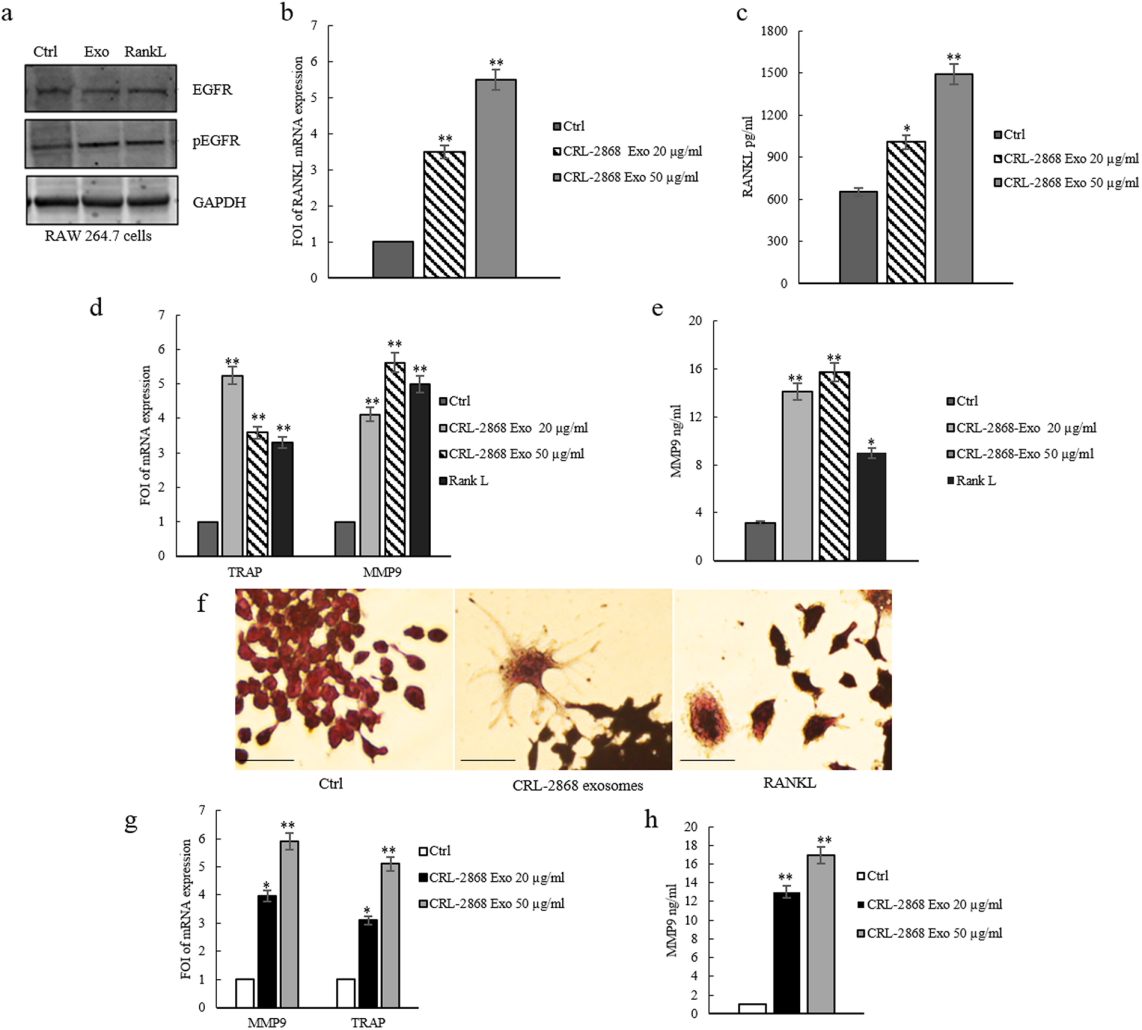
CRL-2868 exosomes induce the activation of EGFR pathway and osteoclast markers gene expression. (**a**) Western blotting analysis of pEGFR and EGFR in whole lysate of RAW 264.7 cells treated, for 6 days, with 20 μg/ml CRL-2868 exosomes (Exo) and RANKL (positive control) compared to untreated cells (Ctrl). Original uncropped WBs were reported in Figure S5C. GAPDH was used as loading control. (**b**) Evaluation by quantitative Real Time PCR of mRNA RANKL expression in RAW 264.7 cells treated, for 6 days, with 20 and 50 μg/ml CRL-2868 exosomes. (**c**) msRANKL protein levels assessed by ELISA, in RAW 264.7 cells treated, for 6 days, with 20 and 50 μg/ml CRL-2868 exosomes. (**d**) Evaluation by quantitative Real Time PCR of mRNA expression of TRAP and MMP9 in RAW 264.7 cells treated, for 6 days, with 20–50 μg/ml CRL-2868 exosomes and RANKL (positive control). (**e**) MMP9 protein level assessed by ELISA, in RAW 264.7 cells treated, for 6 days, with 20–50 μg/ml CRL-2868 exosomes and RANKL. Values are the mean ± SD of 3 three independent experiments *p ≤ 0.05, **p ≤ 0.01. (**f**) TRAP staining of RAW 264.7 cells incubated with CRL-2868 exosomes and 25 ng/ml of RANKL (positive control), for 6 days, compared with untreated cells. Scale bar 10 µm. (**g**) Evaluation by quantitative Real Time PCR of mRNA expression of TRAP and MMP9 in human primary pre-osteoclasts treated, for 4 days, with 20–50 μg/ml of CRL-2868 exosomes. (**h**) MMP9 protein levels assessed by ELISA, in human primary pre-osteoclasts treated, for 4 days, with 20–50 μg/ml CRL-2868 exosomes. Values are the mean ± SD in three independent experiments *p ≤ 0.05, **p ≤ 0.01.

The addition of CRL-2868 exosomes to RAW 264.7 cells induced the activation of EGFR. As shown by western blot analysis, RAW 264.7 cells treated with CRL-2868 exosomes showed an increase of EGFR phosphorylation (Fig. 3a). Densitometric analyses are showed in Figure S2d. We observed an increase of msRANKL gene expression in Raw 264.7 cells treated with CRL-2868 exosomes (Fig. 3b). We obtained similar results after treatment of Raw 264.7 cells with A549 exosomes (Figure S3a). An ELISA assay confirmed that the treatment of Raw 264.7 with CRL-2868 exosomes increased the amount of soluble msRANKL in culture medium (Fig. 3c). Western blot analysis indicated that CRL-2868 exosomes do not contain an estimable amount of EGFR compared to parental cells Figure S2e).

### CRL-2868-exosomes induce osteoclast gene expression

In order to investigate the differentiating effect mediated by EGFR pathway activation, we analyzed the expression of TRAP and MMP9, well-known genes involved in osteoclasts differentiation. CRL-2868 exosomes added to RAW 264.7 cells induced gene expression of TRAP and MMP9 compared to untreated cells (Fig. 3d). Similar results were obtained with RANKL treatment, used as positive control. Moreover, treatment of Raw 264.7 cells with A549 exosomes induced gene expression of TRAP and MMP9 (Figure S3b). We tested, by ELISA assay, MMP9 protein levels in RAW 264.7 cells treated with CRL-2868 exosomes. As shown in Fig. 3e, the treatment with exosomes induced an increase of MMP9 compared to control cells. TRAP staining assay of RAW 264.7 cells treated with CRL-2868 exosomes confirmed the induction of TRAP-positive multinucleate cells (Fig. 3f). We obtained similar results after treatment of Raw 264.7 cells with A549 exosomes (Figure S3c). In order to confirm this mechanism in a human pre-osteoclasts model, we treated human monocytes with CRL-2868 exosomes. CRL-2868 exosomes treatment of human monocytes induced the gene expression of TRAP and MMP9 (Fig. 3g) compared to untreated cells. The ELISA assay for MMP9 confirmed the increased release of metalloprotease in exosome-treated human pre-osteoclasts (Fig. 3h). Furthermore, in order to be sure that CRL-2868 derived exosomes are enriched in AREG, we analyzed the levels of AREG contained in conditioned media exosomes-deprived (CM- Exo). As showed by western blotting CM- Exo did not contain AREG (Figure S4a). We tested the effects of CM- Exo on osteoclast differentiation; we observed that CM- Exo had not effects on RANKL at protein and mRNA levels (Figure S4b and c), MMP9 and TRAP gene expression (Figure S4d) and MMP9 at protein level.

### Erlotinib reverts the effects of NSCLC-exosomes in osteoclast differentiation

**Figure 4:**
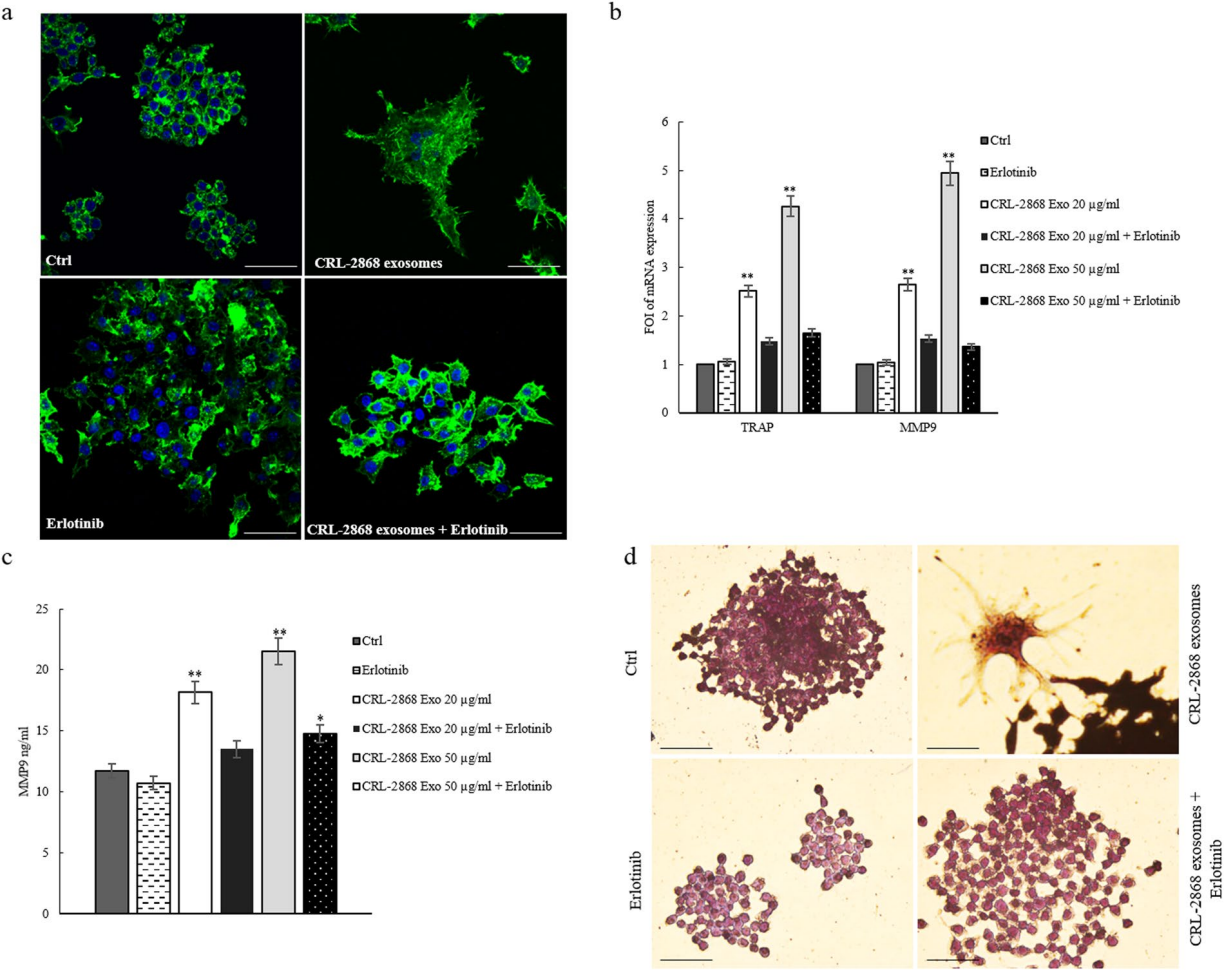
Erlotinib reverts the effects of NSCLC-exosomes in osteoclast differentiation. (**a**) Confocal microscopy analysis of RAW 264.7 cells treated, for 6 days with: CRL-2868 exosomes, Erlotinib and CRL-2868 exosomes plus Erlotinib (CRL-2868 exosomes + Erlotinib) compared with RAW 264.7 control (Ctrl). Scale bar 10 µm. (**b**) Evaluation by quantitative Real Time PCR of mRNA expression of TRAP and MMP9 in RAW 264.7 cells treated, for 6 days, with: 20–50 μg/ml of CRL-2868 exosomes, Erlotinib and CRL-2868 exosomes plus Erlotinib. (**c**) MMP9 protein levels assessed by ELISA, in RAW 264.7 cells treated, for 6 days, with: 20–50 μg/ml of CRL-2868 exosomes, Erlotinib and CRL-2868 exosomes plus Erlotinib. Values are the mean ± SD of three independent experiments *p ≤ 0.05, **p ≤ 0.01. (**d**) TRAP staining of RAW 264.7 cells incubated with: CRL-2868 exosomes, Erlotinib, and CRL-2868 exosomes plus Erlotinib, for six days, compared with untreated RAW 264.7 cells. Scale bar 10 µm.

Furugaki and colleagues have demonstrated that Erlotinib inhibits tumor-induced osteolytic bone metastasis by suppressing osteoclast activation through (i) the inhibition of tumor growth at the bone metastatic sites, (ii) the osteolytic factor production in tumor cells, (iii) the osteoblast proliferation and osteoclast differentiation from mouse bone marrow cells^17^. We observed that in RAW 264.7 cells the co-treatment with CRL-2868 exosomes and Erlotinib reverted the effects of exosomes in osteoclastogenesis. As shown in Fig. 4a, the co-treatment with CRL-2868 exosomes and Erlotinib reverted morphological RAW 264.7 cells differentiation in mature osteoclasts. The co-treatment also inhibited CRL-2868 exosomes-induced expression of MMP9 (Fig. 4b and c) and TRAP (Fig. 4b and d) at mRNA and protein level. We obtained similar results after treatment of Raw 264.7 cells with A549 exosomes (Figure S3a, b and c).

### Amphiregulin contained in NSCLC-exosomes induces osteoclast differentiation

**Figure 5:**
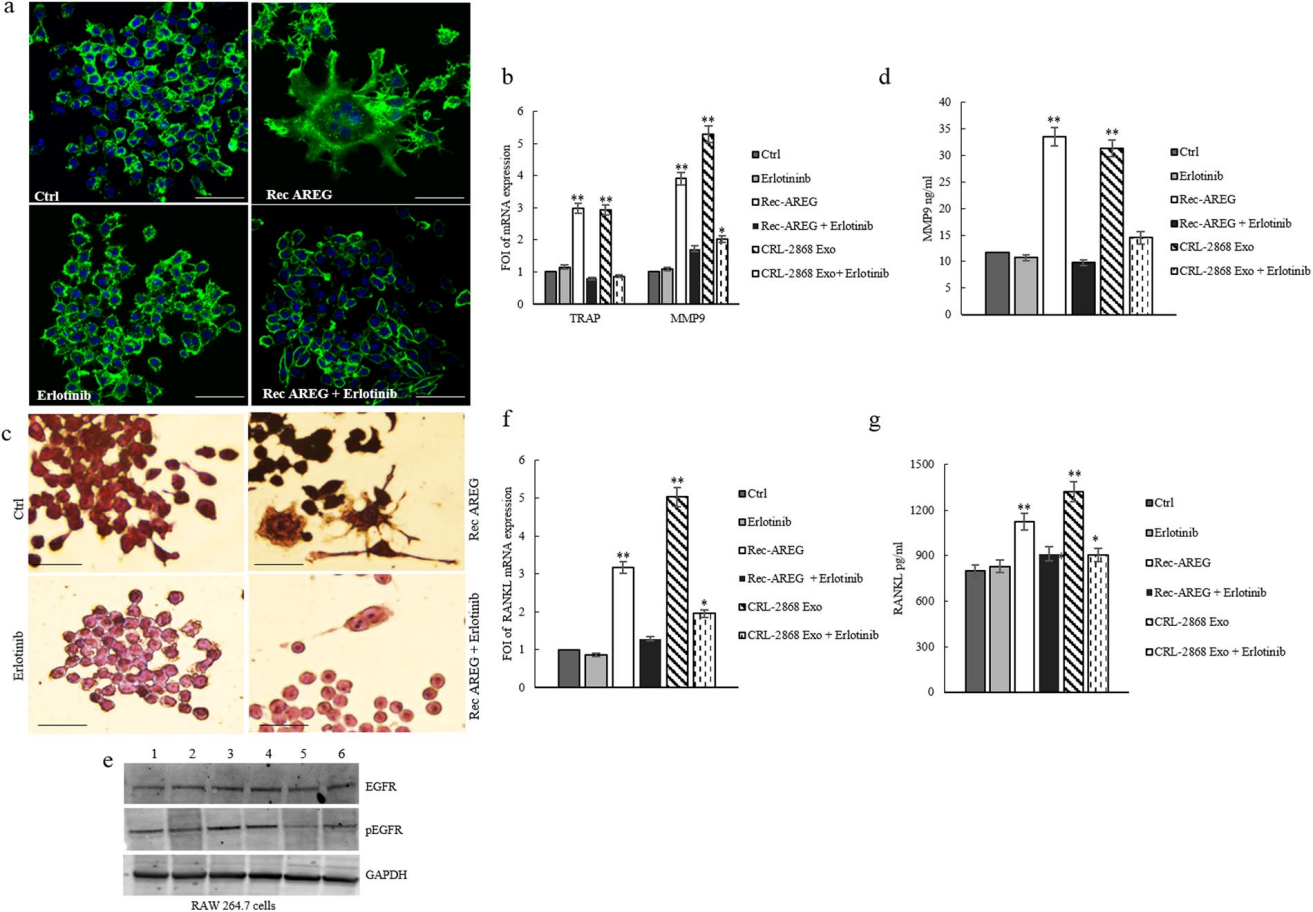
Rec-AREG induces osteoclast differentiation. (**a**) Confocal microscopy analysis of RAW 264.7 cells treated, for 6 days with: CRL-2868 exosomes, Rec-AREG and Rec-AREG plus Erlotinib (Rec-AREG + Erlotinib) compared with untreated RAW 264.7 cells (Ctrl). Scale bar 10 µm. (**b**) Evaluation by quantitative Real Time PCR of mRNA expression of TRAP and MMP9 in RAW 264.7 cells treated, for 6 days, with: 20–50 μg/ml of CRL-2868 exosomes, Rec-AREG and Rec-AREG plus Erlotinib (Rec-AREG + Erlotinib). (**c**) TRAP staining of RAW 264.7 cells incubated with: CRL-2868 exosomes, Rec-AREG, Erlotinib, CRL-2868 exosomes plus Erlotinib (CRL-2868 Exo + Erlotinib), Rec-AREG plus Erlotinib (Rec-AREG + Erlotinib), for 6 days, compared with untreated RAW 264.7 cells. (**d**) MMP9 protein level assessed by ELISA, in RAW 264.7 cells treated, for 6 days, with: CRL-2868 exosome, Rec-AREG, CRL-2868 exosomes plus Erlotinib (CRL-2868 Exo + Erlotinib), Rec-AREG plus Erlotinib (Rec-AREG + Erlotinib). Values are the mean ± SD of three independent experiments *p ≤ 0.05, **p ≤ 0.01. (**e**) Western blotting analysis of pEGFR and EGFR in whole lysate of RAW 264.7 cells treated, for 6 days, with: Erlotinib (2), Rec-AREG (3), CRL-2868 exosomes (4), Rec-AREG plus Erlotinib (5), CRL-2868 exosomes plus Erlotinib (6) compared to untreated cells (1). GAPDH was used as loading control Original uncropped WBs were reported in Figure S5D. (**f**) Evaluation by quantitative Real Time PCR of mRNA expression of mRNA RANKL expression in RAW 264.7 cells treated, for 6 days, with: CRL-2868 exosomes, Rec-AREG, CRL-2868 exosomes plus Erlotinib (CRL-2868 Exo + Erlotinib), Rec-AREG plus Erlotinib (Rec-AREG + Erlotinib). (**g**) RANKL protein levels assessed by ELISA, in RAW 264.7 cells treated, for 6 days, with: CRL-2868 exosomes, Rec-AREG, CRL-2868 exosomes plus Erlotinib (CRL-2868 Exo + Erlotinib), Rec-AREG plus Erlotinib (Rec-AREG + Erlotinib). Values are the mean ± SD of 3 three independent experiments *p ≤ 0.05, **p ≤ 0.01.

In order to analyze if exosomal AREG induced EGFR pathway activation causing osteoclast differentiation, Raw 264.7 cells were first treated with recombinant AREG (Rec-AREG). As demonstrated by confocal analyses (Fig. 5a), addition of Rec-AREG to RAW 264.7 cells induced the expression of the osteoclast differentiation markers as also demonstrated by the increased expression of TRAP (Fig. 5b and c) and MMP9 (Fig. 5b and d) at mRNA and protein level. These effects reverted after co-treatment with Rec-AREG and Erlotinib (Fig. 5b–e), further supporting the involvement of EGFR pathway in osteoclastogenesis.

Moreover, the treatment of RAW 264.7 cells with Rec-AREG induced an increase of EGFR phosphorylation (Fig. 5e), that in turn enhanced the expression of msRANKL at mRNA and protein levels (Fig. 5f and g).

As shown by western blotting analysis, in RAW 264.7 cells the co-treatment with CRL-2868 exosomes and Erlotinib reverted the increase of EGFR phosphorylation induced by exosomes (Fig. 5e). Densitometric analyses are showed in Figure S2d.

### Knockdown of AREG in CRL-2868 cells causes a decrease of exosomal AREG and Neutralizing AREG antibodies revert the effects of CRL-2868 exosomes

**Figure 6:**
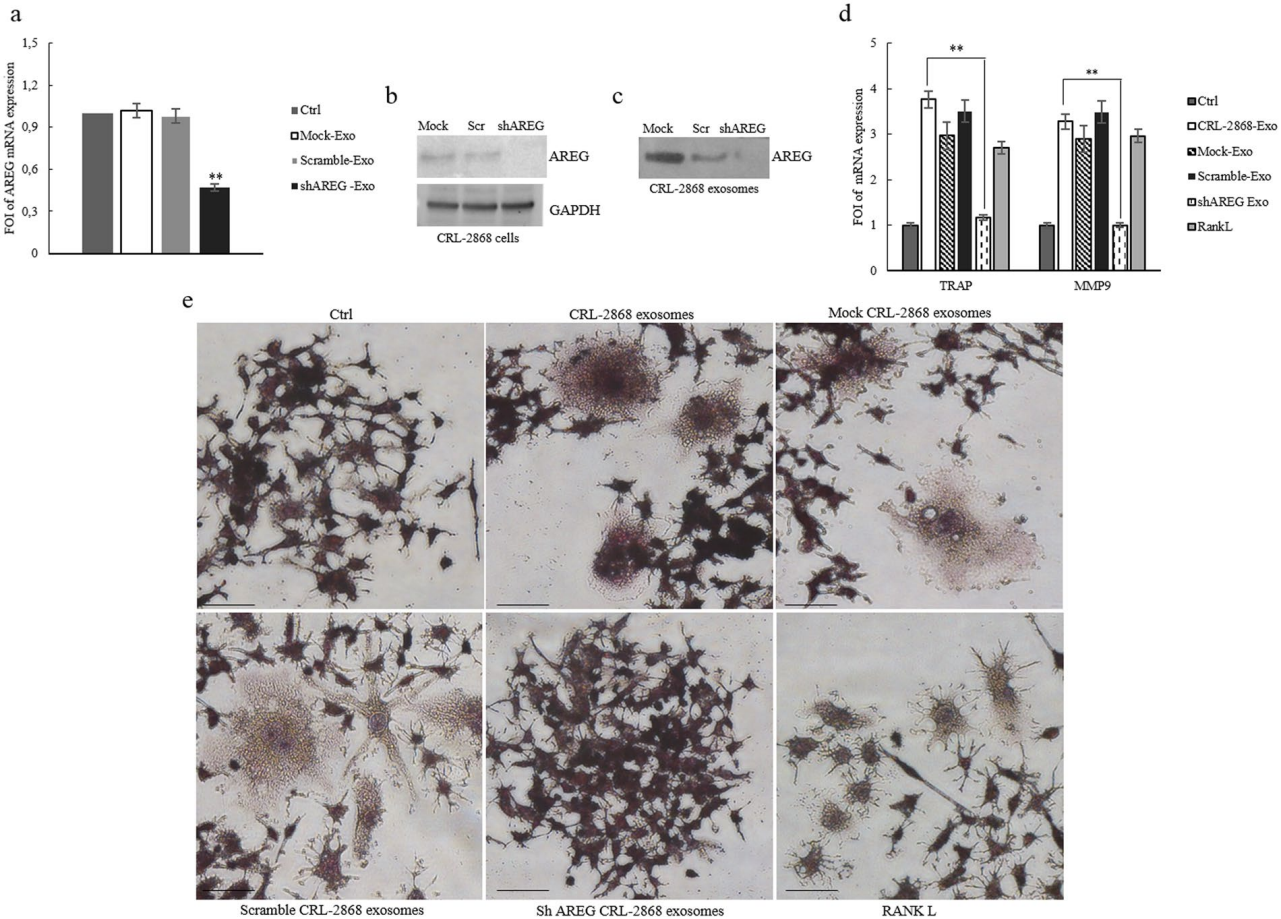
Knockdown of AREG in CRL-2868 cells. (**a**) Evaluation by real time PCR analysis of mRNA expression of AREG in CRL-2868 cells transfected with AREG shRNA plasmid (AREG-knockdown CRL-2868 cells), empty vector (Mock-CRL-2868 cells) and control AREG shRNA plasmid (Scramble-CRL-2868 cells). (**b**) Western blotting of AREG in whole lysate of Mock, Scramble, and AREG-knockdown CRL-2868 cells. GAPDH was used as loading control. Original uncropped WBs were reported in Figure S5E. (**c**) Western blotting of AREG in exosomes released by Mock, Scramble, and AREG-knockdown CRL-2868 cells. Original uncropped WBs were reported in Figure S5E. (**d**) Evaluation by real time PCR analysis of mRNA expression of TRAP and MMP9 in RAW 264.7 cells treated, for 6 days, with exosomes released by Control, Mock, Scramble, and AREG-knockdown CRL-2868 cells and RANKL. Values are the mean ± SD of three independent experiments *p ≤ 0.05, **p ≤ 0.01. (**e**) RAW 264.7 cells were incubated with exosomes released by Control, Mock, Scramble, and AREG-knockdown CRL-2868 cells and RANKL, for 6 days, stained for TRAP and compared with untreated cells (Ctrl).

According to the results described so far, our data indicated that elevated levels of AREG in NSCLC-exosomes induce EGFR pathway activation thus promoting osteoclast differentiation. In order to confirm our hypothesis, we reduced the AREG level in the CRL-2868 cells by stably expressing human AREG shRNA. Following puromycin (4 μg/mL) selection, we isolated AREG shRNA cells (AREG-knockdown CRL-2868 cells) and compared them with cells transfected with empty vector (Mock-CRL-2868 cells) and control AREG shRNA plasmid (Scramble-CRL-2868 cells). The expression levels of AREG decreased in AREG shRNA cells with respect to control cells as shown by real time PCR (Fig. 6a) and western blot analysis (Fig. 6b). We also found a strong decrease of AREG levels in CRL-2868 exosomes from AREG-knockdown CRL-2868 cells, as showed by western blotting (Fig. 6c).

In RAW 264.7 cells the treatment with exosomes released by AREG-knockdown CRL-2868 cells did not affect the expression of the osteoclast differentiation markers as demonstrated by the levels of TRAP and MMP9 mRNA (Fig. 6d). Furthermore, as shown in Fig. 6e in Raw 264.7 cells the treatment with exosomes released by CRL-2868 cells Control, Mock and Scramble induced the formation of TRAP-positive multinucleate cells compared to untreated RAW 264.7 cells or incubated for six days with exosomes released by AREG-knockdown CRL-2868 cells. These data confirm the role played by AREG in osteoclast differentiation.

**Figure 7:**
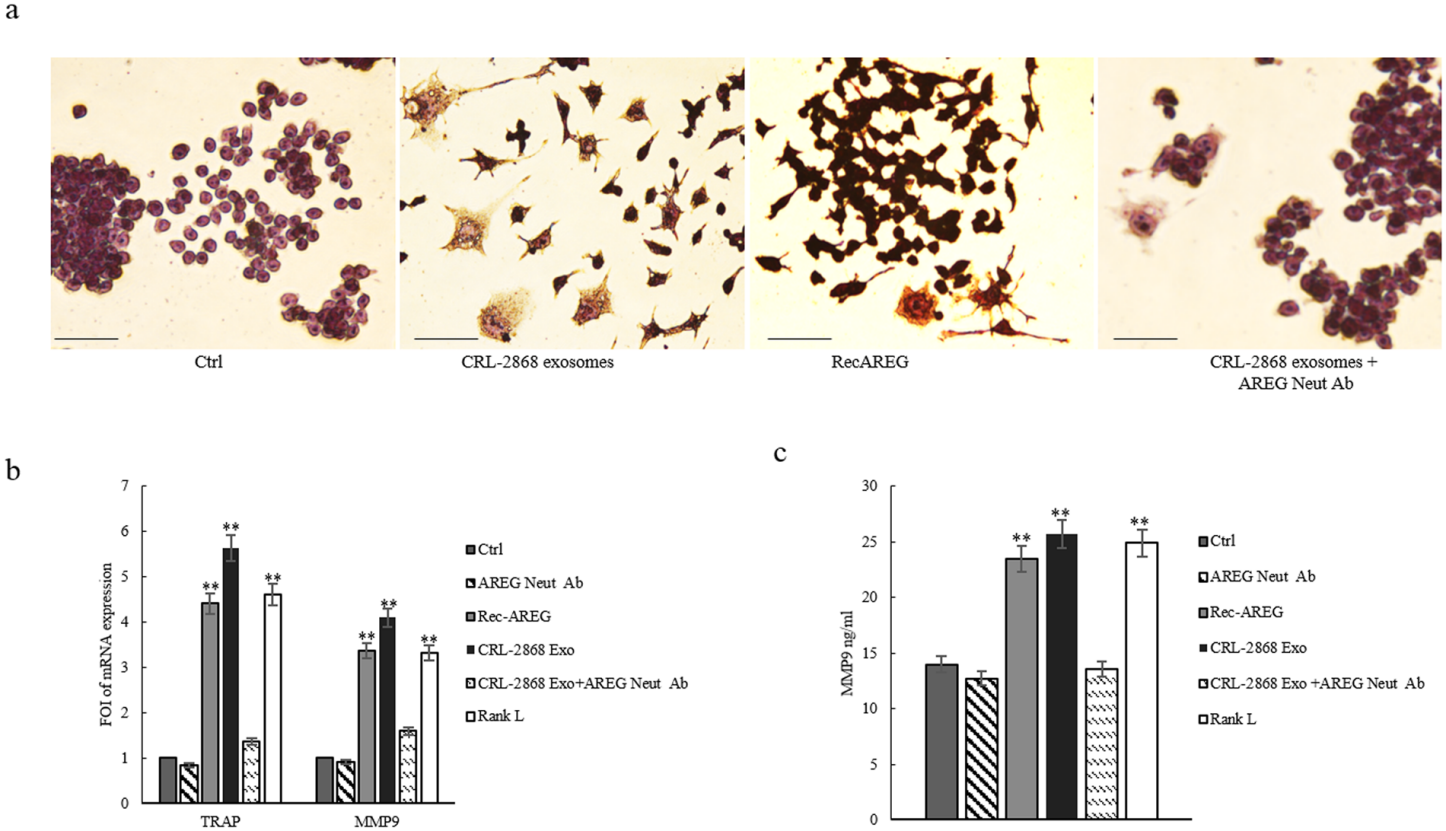
Neutralizing AREG antibodies revert osteoclast differentiation induced by NSCLC-exosomes. (**a**) TRAP staining of RAW 264.7 cells incubated with: CRL-2868 exosomes, Rec-AREG, CRL-2868 exosomes plus AREG neutralizing antibodies, for 6 days, stained for TRAP and compared with untreated cells (Ctrl). Scale bar 10 µm. (**b**) Evaluation by real Time PCR analysis of mRNA expression of TRAP and MMP9 in RAW 264.7 cells treated, for 6 days, with: CRL-2868 exosomes, Rec-AREG, AREG neutralizing antibodies, RANKL, CRL-2868 exosomes plus AREG neutralizing antibodies. (**c**) MMP9 protein levels assessed by ELISA, in RAW 264.7 cells treated, for 6 days, with: CRL-2868 exosomes, Rec-AREG, AREG neutralizing antibodies, RANKL, CRL-2868 exosomes plus AREG neutralizing antibodies. Values are the mean ± SD of three independent experiments *p ≤ 0.05, **p ≤ 0.01.

The co-treatment of RAW 264.7 with CRL-2868 exosomes and AREG neutralizing antibodies reverted the effects on osteoclast differentiation mediated by exosomes. As shown in Fig. 7a, the co-treatment caused a decrease of a number of TRAP positive multinucleate osteoclasts and of TRAP and MMP9 (Fig. 7b) expression compared to the treatment with exosomes alone. An ELISA assay for MMP9 confirmed that the co-treatment with CRL-2868 exosomes and AREG neutralizing antibodies reverted the effects on osteoclast differentiation mediated by exosomes (Fig. 7c). We obtained similar results after treatment of Raw 264.7 cells with A549 exosomes (Figure S3a, b and c).

### Exosomes released in plasma of NSCLC patients modulate osteoclastogenesis in human primary osteoclasts

**Figure 8:**
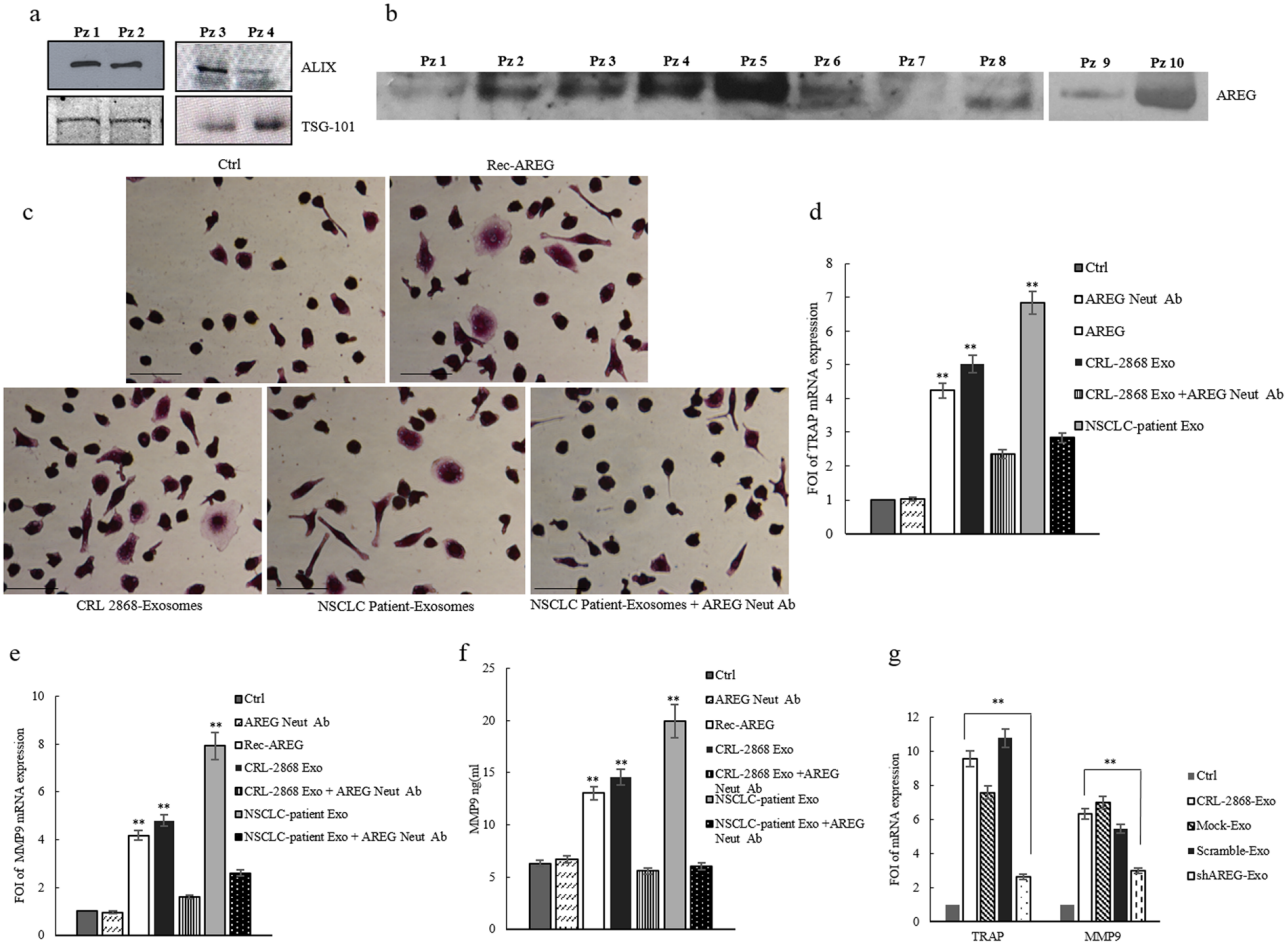
Exosomes from NSCLC plasma patients modulate osteoclastogenesis in human primary osteoclasts. (**a**) Representative western blotting of ALIX and TSG 101 in 30 µg of exosomes isolated from plasma (1, 5 ml) of four NSCLC patients (Pz 1:stage I, Pz 2:stage IB, Pz 3:stage IIIA, Pz 4:stage IIIA). Original uncropped WBs were reported in Figure S5F. (**b**) Representative western blotting of AREG in 30 µg of exosomes isolated from plasma of ten NSCLC patients at different disease stages (Pz 1:stage I, Pz 2:stage IB, Pz 3:stage III, Pz 4:stage IIIA, Pz 5:stage IV, Pz 6:stage IV, Pz 7:stage IV, Pz 8:stage IV, Pz 9:stage IV, Pz 10:stage IV). Original uncropped WBs were reported in Figure S5G. (**c**) TRAP staining of human pOCs cultured in differentiation medium and incubated with: REC-AREG, CRL-2868 exosomes, NSCLC-patient exosomes, NSCLC-patient exosomes plus AREG neutralizing antibodies. Scale bar 10 µm. Evaluation by Real Time PCR of TRAP (**d**) and MMP9 (**e**) mRNA expression, in primary human pre-osteoclasts, treated for 4 days, with: CRL-2868 exosomes, AREG neutralizing antibodies, NSCLC patient exosomes, CRL-2868 exosomes plus AREG neutralizing antibodies, NSCLC patients exosomes plus AREG neutralizing antibodies. (**f**) MMP9 protein levels assessed by ELISA, in primary human pre-osteoclasts treated for 4 days with: CRL-2868 exosomes, AREG neutralizing antibodies, NSCLC patient exosomes, CRL-2868 exosomes plus AREG neutralizing antibodies, NSCLC patients exosomes plus AREG neutralizing antibodies. (**g**) Evaluation by real time PCR analysis of mRNA expression of TRAP and MMP9 in primary human pre-osteoclasts treated for 4 days with: exosomes released by Control, Mock, Scramble, and AREG-knockdown CRL-2868 cells. Values are the mean ± SD of three independent experiments *p ≤ 0.05, **p ≤ 0.01.

In order to confirm *ex vivo* the effects of exosomal AREG on osteoclastogenesis, human primary osteoclasts were treated with exosomes released in plasma of NSCLC patients. Extracellular vesicles from NSCLC patients were characterized by Western blotting using antibodies specific for ALIX, TSG 101 to confirm their exosomal identity (Fig. 8a). Exosomes released in plasma of twenty NSCLC patients at different disease stage contained AREG, as showed by a representative western blotting of exosomes purified from ten samples of NSCLC plasma patients (Fig. 8b).

Human PBMCs were treated with RANKL and dexamethasone, after 4 days, the adherent cells were mononucleated, expressed TRAP and considered committed pre-osteoclast cells (POs). POs were treated, for 4 days, with exosomes from plasma of NSCLC patients as shown in Fig. 8c, POs acquired the morphology of mature osteoclasts. The treatment of POs with exosomes released in plasma of NSCLC patients induced an increase of TRAP (Fig. 8c and d) and MMP9 (Fig. 8e and f) at mRNA and protein levels. The same effects were obtained following Rec-AREG treatments of POs. The effects on osteoclast differentiation mediated by NSCLC exosomes reverted after cotreatment with AREG neutralizing antibodies. Furthermore, human monocytes were treated with CRL-2868 exosomes released by AREG-knockdown CRL-2868 cells compared to POs treated with exosomes released by CRL-2868 cells Control, Mock and Scramble. The treatment with exosomes released by AREG-knockdown CRL-2868 cells of human monocytes did not increase of TRAP and MMP9 levels as occurred with exosomes isolated from Control, Mock and Scramble CRL-2868 cells (Fig. 8g).

Overall, these data supported the hypotheses that AREG contained in the membranes of exosomes isolated from CRL-2868 cell conditioned medium induced EGFR phosphorylation. EGFR pathway activation was able to increase RANKL expression, by osteoclast precursor, that in turn induced MMP9 and TRAP expression, well known markers of osteoclast differentiation.

## Discussion

The major sites of NSCLC metastases include brain, bone, liver, thoracic cavity and distant lymph nodes^25–27^. Bone microenvironment can promote the growth of lung cancer metastases through the interaction of metastatic cells with osteoclasts and osteoblasts promoting bone degradation that in turn causes the release of ECM bound growth factors^28, 29^. Osteolytic bone metastasis frequently occurs in the later stages of several cancers^30^. Multiple myeloma (MM) and breast cancer induce lytic lesions and have been shown to exhibit high levels of RANKL and low levels of OPG^31, 32^. The unbalance between RANKL and OPG contributes to NSCLC development *in vivo* ^6^; in xenograft mice model has been demonstrated that RANKL overexpression promoted bone destruction and tumor growth of NSCLC cells^18^.

Furthermore, it was demonstrated that EGFR regulates osteoclast differentiation through the crosstalk with RANK signaling^8^. EGFR is expressed in osteoclast lineage cells and RANKL-mediated osteoclastogenesis requires intact EGFR signaling. Interestingly, EGFR-deficient mice showed defective osteoclast recruitment^33^.

Exosomes released by cancer cells prepare an opportune microenvironment at future metastatic sites and mediate non-random patterns of metastasis^15, 16, 34^. Recently our research group showed that MM exosomes had a role in osteoclast differentiation^20^. Now, this work demonstrates that AREG contained in NSCLC-exosomes plays a key role in the induction pre-osteoclast differentiation in mature osteoclasts. Exosomes released in CRL-2868 conditioned media (Fig. 1) and in plasma of NSCLC patients (Fig. 8) were isolated and characterized through morphological and biochemical analyses.

In line with data from literature, we showed that RAW 264.7 cells treated with NSCLC exosomes activate EGFR pathway that caused an upregulation of RANKL (Fig. 3) and of osteoclastogenesis markers (MMP9 and TRAP). In order to confirm the central role of EGFR pathway activation in the induction of osteoclastogesis, we tested the effects of Erlotinib in the osteoclasts differentiation mediated by NSCLC exosomes. The co-treatment of pre-osteoclasts with exosomes and Erlotinib reverted the effect of exosomes (Fig. 4) in osteoclasts differentiation, indicating that the block of EGFR pathway inhibited osteoclastogenesis. Tyrosine kinase inhibitors (TKIs) of EGFR activity have been introduced several years ago to treat NSCLC patients. Erlotinib, one of the first-generation EGFR-TKIs^35–37^ binds competitively and reversibly to the ATP-binding site of the EGFR TK domain, and shows a significant advance treatment in selected NSCLC patients with activating EGFR mutations. Recent data reported that exosomes released by colon and breast cancer cells contained the EGFR-ligand, Amphiregulin, and AREG-exosomes increased invasiveness of recipient cancer cells^10^. Our research group also demonstrated that AREG was contained in exosomes released by chronic myeloid leukemia cells and contributed to establish a bidirectional crosstalk between leukemic and stromal cells. Exosomal AREG promoted proliferation and survival of leukemic cells, both *in vitro* and *in vivo*, inducing IL8 secretion from stromal cells^38, 39^. AREG has been identified in serum of NSCLC patients; Ishikawa and colleagues suggested that circulating Amphiregulin and TGF-α could be clinically applicable as indicators for an unfavorable response to Gefitinib by identifying patients with a higher probability of drug resistance^40^.

In order to test if exosomal AREG was an important molecule in the induction of EGFR pathway, we performed experiments with recombinant AREG and AREG neutralizing antibody. As showed in Fig. 6, the treatment of pre-osteoclasts with recombinant AREG had the similar effects of NSCLC-exosomes on the induction of EGFR phosphorylation causing an increase of RANKL that modulated MMP9 and TRAP expression and induced the typical phenotype of mature osteoclasts. The co-treatment of pre-osteoclasts with NSCLC-exosomes and AREG neutralizing antibodies reverted the effects of osteoclasts differentiation mediated by NSCLC exosomes (Fig. 7).

The central role of exosomal AREG in osteoclast differentiation was confirmed by a knockdown of AREG in CRL-2868 cells. The decrease of AREG levels in CRL-2868 cells inhibited the accumulation of this molecule into exosomes, reverting the effects on osteoclastogenesis induced by lung cancer exosomes (Fig. 6). The data obtained in Raw 264.7 cells treated with exosomes released by CRL-2868 cell line were confirmed with human committed preosteoclast PMBCs treated with esosomes isolated from plasma of twenty NSCLC patients at different disease stages. We demonstrated that NSCLC patient exosomes were enriched in AREG and they induced the human preosteoclast differentiation in mature osteoclasts. These effects reverted after treatment with AREG neutralizing antibodies (Fig. 8). However, despite described correlation between increased amounts of AREG in NSCLC^9, 41^ sera with poor prognosis we did not find the same correlation with exosomal AREG and NSCLC stadiation. Probably, constitutive EGFR activation in NSCLC cells leads to an increase of AREG signaling since the early disease stage, driving the cancer progression toward osteolytic bone metastasis.

**Figure 9:**
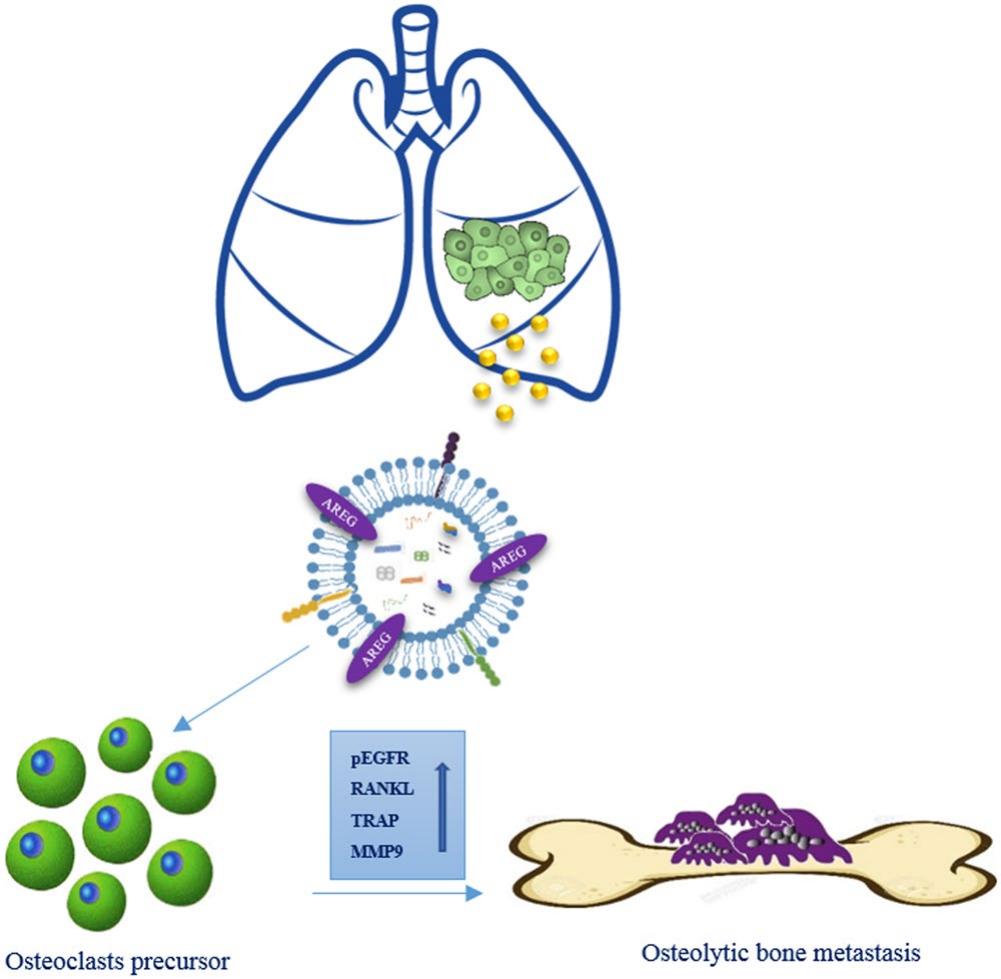
Working hypothesis of the effects of NSCLC exosomes in osteoclast differentiation. CRL-2868 cells released exosomes enriched in AREG that activating EGFR pathway in a pre-osteoclast model induced morphological differentiation and RANKL expression at mRNA and protein levels that in turn is able to increase the expression of TRAP and MMP9 well-known markers of osteoclastogenesis.

Taken together our data indicated that exosomal AREG induces the activation of EGFR pathway that increases, in pre-osteoclasts treated with NSCLC-exosomes, the expression of RANKL at mRNA and protein levels. RANKL, in turn is able to induce the expression of proteolytic enzymes considered osteoclastogenesis markers, triggering the vicious cycle (Fig. 9). Shed light on the role of AREG contained in NSCLC-exosomes in the osteoclast differentiation might permit to improve the therapeutic strategy to inhibit the fatal attraction between lung cancer and bone.

## Methods

### Cell lines and reagents

Non-small cell lung cancer, CRL-2868 cell line was obtained from Prof Christian D. Rolfo (University Hospital Antwerp Oncology). CRL-2868 cells were grown in RPMI-1640 (Euroclone, UK) supplemented with 10% Fetal Bovine Serum (FBS, Euroclone, UK), 100 U/ml penicillin and 100 µg/ml streptomycin (Euroclone, UK) and with 1% Sodium pyruvate (Euroclone, UK). Non-small cell lung cancer, A549 cell line and prostate cancer, PC3 cell line were obtained from ATCC and were grown in RPMI-1640 (Euroclone, UK) supplemented with 10% Fetal Bovine Serum (FBS, Euroclone, UK), 100 U/ml penicillin and 100 µg/ml streptomycin (Euroclone, UK). Breast cancer, MDA-MB-231 cell line was obtained from ATCC and grown in DMEM-F12 (Euroclone, UK) supplemented with 10% Fetal Bovine Serum (FBS, Euroclone, UK), 100 U/ml penicillin and 100 µg/ml streptomycin (Euroclone, UK).

Murine macrophage Raw 264.7 cells were purchased from ATCC^®^ and cultured in Dulbecco’s modified Eagle’s medium (DMEM), supplemented with 10% FBS, 100 U/ml penicillin and 100 µg/ml streptomycin. To induce differentiation, RAW 264.7 cells were treated with 25 ng/ml of human recombinant RANK Ligand (RANKL) (Gibco, Life Techonologies, USA) for 6 days in DMEM, supplemented with 10% FBS, previously ultracentrifugated (OC medium). Alternately, cells were treated for 6 days with 20 µg/ml and 50 µg/ml of CRL-2868 cell-derived exosomes (CRL-2868-exosomes), in DMEM, supplemented with 10% of ultracentifugated FBS (see below). Erlotinib (Cayman Chemical, Ann Arbor, MI, USA) was solubilized at 10-mM stock solution in DMSO and stored at −20 °C. Neutralizing antibody anti-AREG (R&D Systems, Abingdon, UK) was reconstituted at 0.2 mg/ml in sterile PBS, aliquoted and stored at −20 °C. Recombinant AREG (R&D Systems, Abingdon, UK) was reconstituted at 0.1 mg/ml in sterile PBS, aliquoted and stored at −20 °C. Working dilutions, where necessary, were prepared in medium.

### Isolation of human peripheral blood mononuclear cells

Human blood samples were obtained from two healthy donors, after written informed consent obtained in accordance with the Declaration of Helsinki guidelines and Antwerp University Ethics committee n. 14/17/206. Human peripheral blood mononuclear cells (PBMCs) were isolated using the Ficoll-Paque (GE Healthcare Bio Science, Uppsala, Sweden) separation technique.

### Preparation of human primary preosteoclasts (pOCs) and osteoclasts (OCs)

PBMCs were cultured in 12-well plates at 1.5 × 10^6^ cells per well in RPMI-1640 supplemented with 10% FBS previously ultracentrifuged, 25 ng/ml of human recombinant RANK Ligand, 25 ng/ml of human MCSF (Gibco, Life Technologies, USA), and 10 nM dexamethasone (Sigma-Aldrich Italy) (Human OC medium). After 2–4 days, the culture were washed with RPMI-1640 medium to remove non-adherent cells. The adherent cells were mononucleated expressed TRAP and were considered committed pre-osteoclast. For human osteoclastogenesis assays, OC medium was added and the cultures were continued for additional 4 days, at the end of the incubation, they contained large mature multinucleated OCs. The culture period was 6–8 days for both TRAP staining assay and qRT-PCR analysis.

### NSCLC patients

Exosomes isolated from human blood samples were obtained from twenty diagnosed NSCLC patients at different disease stages. Informed consent was obtained from patients, according to the Declaration of Helsinki and with hospital ethics committee approval (Antwerp University Ethics committee n. 14/17/206). Plasma patients (NSCLC) derived exosomes were isolated as described in the section “Exosomes isolation”. The patient population was selected according to these inclusion criteria: histologically or cytologically confirmed diagnosis of stage III or IV, EGFR mutated NSCLC; presence or not of bone metastasis; age 18 years or older at the time of informed consent; smokers and no-smokers declaration, sensitive to first line TKI inhibitors.

### Exosomes isolation

Exosomes released by CRL-2868 cells after a 24 hours culture period in the presence of FBS previously ultracentrifugated (vesicle-free media) were isolated from conditioned culture medium by differential centrifugation as described by Thery *et al*. (1). Exosomes pellet was washed and then resuspended in PBS. In average, we obtained 20 µg of exosomes from 7, 5 × 10^5^ cells and 50 µg of exosomes from 1, 8 × 10^6^ cells. The same exosomes isolation procedure was used for the other cell lines. Exosome protein content was determined by the Bradford assay (Pierce, Rockford, IL, USA). Exosomes characterization was previously described by our group (2). Briefly, exosomes from plasma of NSCLC patients (1, 5 ml) were isolated after the 100,000 × g ultracentrifugation for 1 hour and 45 minutes at 4 °C to pellet the exosomes. Exosomes pellet was washed and then resuspended in PBS. Exosomes protein content was determined by the Bradford assay.

### Uptake of CRL-2868-exosomes by Raw264.7 cells

CRL-2868 and A549 cell-derived exosomes are labeled with PKH26 (Sigma-Aldrich, Italy), according to the manufacturer’s instructions. Briefly, exosomes collected after the 100,000 × g ultracentrifugation, were incubated with PKH26 for 10 min at room temperature. Labeled exosomes were washed in PBS, centrifugated and resuspended in low serum medium and incubated with Raw 264.7 cells seeded in 24-well plates at a density of 100.000 cells per well for 1–3 hours at 37 °C. In a set of experiments, RAW 264.7 cells were pretreated with 50 µM 5-ethyl-N-isopropyl amiloride (EIPA), a known inhibitor of exosomes uptake, for 3 h. After incubation, cells were processed as previously described (2). RAW 264.7 cells were stained with ActinGreen^TM^ 488 Ready Probes^R^ Reagent (Life Technologies, USA) that binds F-actin with high affinity. Nuclei were stained with Hoechst (Molecular Probes, Life Technologies, USA) and analyzed by confocal microscopy (Nikon Eclipse T*i*).

### Transmission electron microscopy (TEM) of Raw 264.7 cells

Murine macrophage RAW 264.7 cells were grown under the same conditions as previously described, for seven days. After a brief rinse in PBS, they were fixed with 2.5% glutaraldehyde 0.1 M sodium cacodylate, pH 7.3, for 60 min at room temperature. Cells were then rinsed, post-fixed with 1% osmium tetroxide in the same buffer for 1 h at 4 °C, and dehydrated in ascending alcohols. After embedding in Epon resin, ultrathin sections were cut using a Reichert Ultracut E, stained, and examined using a transmission electron microscope (Jeoll JEM-1400 Plus,), at 80 kV. Images were taken with Digital CCD Camera 8 M.

### Transmission electron microscopy of CRL-2868 exosomes

For electron microscopic studies, a 5 μl aliquot of exosome preparations was placed onto carbon-coated 200-mesh copper grids (Electron Microscopy Sciences, USA) for 20 min at room temperature. After, the samples were fixed for 5 min in 1% glutaraldehyde in PBS and negatively stained with 2% aqueous solution of phosphotungstic acid. The grids were examined using JEOL JEM-1400 Plus electron microscope, at 80 kV.

### Immunoelectron microscopy

Membrane vesicles were resuspended and applied onto 200-mesh grids with form var- carbon-coating. The samples were blocked with 1% bovine serum albumin in PBS. The grids were incubated with rabbit Ab against AREG, followed by goat anti-rabbit IgG coupled to 12-nm gold (Jackson). Control grids were also included, in which the primary antibody was omitted. The grids were post-fixed in 1%glutaraldehyde in PBS, negatively contrasted and examined under Jeoll JEM-1400 Plus electron microscope.

### Knockdown of Amphiregulin with shRNA Plasmid in CRL-2868 cells

Stable transfection of Amphiregulin shRNA Plasmid sc-39412-SH (Santa Cruz Biotechnology, Inc.) in CRL 2868 cells was carried out according to the suggestions from Santa Cruz Biotechnology, Inc. Cell line cultures were grown in a six well tissue culture plate, in RPMI medium supplemented with 10% FBS (without standard antibiotics) to reach about 50–70% confluence of well. The optimal shRNA Plasmid DNA: shRNA Plasmid Transfection Reagent ration, experimentally determined is 3 µg of shRNA Plasmid DNA and 3 µl of shRNA Plasmid Transfection Reagent. Following incubation for 30 minutes at room temperature, the shRNA Plasmid DNA/ShRNA Plasmid Transfection Reagent Complex were added to CRL 2868 cells. Following incubation for 7 hours at 37 °C in a CO2 incubator, RPMI medium containing 2 times the normal serum and antibiotics concentration (2x normal growth medium) was added. The cells were incubated for an additional 24 h at 37 °C in a CO2 incubator. For selection of stably transfected cells selective antibiotic puromycin, at the concentration of 4 µg/ml, was added to the medium 48 hours post-transfection. Every 2 days the growth medium was aspirated and replaced with freshly prepared selective media. Controls shRNA Plasmids included: Control shRNA Plasmid-A (sc-108060), Control shRNA Plasmid-B (sc-108065), Control shRNA Plasmid-C (sc-108066) and cofGFP Control Plasmid (sc-108083).

### TRAP staining assay

RAW 264.7 cells and human primary osteoclasts were stained for detection of tartrate-resistant acid phosphatase (TRAP) activity according to the manufacturer’s protocol (Acid Phosphatase, Leukocyte (TRAP) Kit; Sigma–Aldrich, USA) and evaluated by optical microscopy. Raw 264.7 were seeded in 12-well plates at a density of 2.000 cells per well and treated for 6 days with CRL-2868 exosomes (20–50 μg/ml), A549 exosomes (20–50 μg/ml), or recombinant AREG (20–50 ng/ml) or RANK Ligand 25 ng/ml ± Erlotinib 0.5 µM. Neutralizing antibody anti-AREG (20 ng/ml) was incubated with CRL-2868-exosomes or A549 exosomes (50 µg/ml) for 1 h at 37 °C and used to treat RAW 264.7 cells for 6 days. Human primary osteoclasts were seeded in 12-well plates at a density of 1.5 × 10^6^ cells per well and cultured in OC medium alone or with CRL-2868-exosomes (20–50 µg/ml) ± Erlotinib 0.5 µM or with plasma patients (NSCLC) derived exosomes (20 µg/ml). TRAP positive multinucleated cells were scored as mature osteoclasts. Three independent experiments were performed in triplicate; cells from five different fields were counted for each condition.

### ELISA assay

Briefly, MMP9 levels secreted by both Human primary OCs and Raw264.7 cells were quantified respectively by Human MMP-9 ELISA assays (Invitrogen) and mouse ELISA Kit for MMP9 Cloud-clone Corp®. RANK Ligand levels secreted by both Human primary OCs and RAW 264.7 cells were quantified respectively by ELISA Complete kit human sRANKL assay (KOMABIOTECH) and ELISA Complete kit mouse sRANKL assay (KOMABIOTECH). For details, see supplemental data.

### Statistical analysis

Data were expressed as mean ± SEMs of three independent experiments. Statistical analysis was performed by using an unpaired Student’s *t*-test. Differences were considered to be significant when *P* values were smaller than 0.05.

## Electronic supplementary material

Supplementary data
