# Supplementary material for: Amphiregulin contained in NSCLC-exosomes induces osteoclast differentiation through the activation of EGFR pathway
Source: Sci Rep. 2017 Jun 9;7:3170. doi: 10.1038/s41598-017-03460-y (PMC5466625; doi:10.1038/s41598-017-03460-y)
Supplement: Supplementary file 1 — Supplementary data [file 41598_2017_3460_MOESM1_ESM.doc]

**Supplementary information**

**Amphiregulin contained in NSCLC-exosomes induces osteoclast differentiation through the activation of EGFR pathway**

*Simona Taverna^1,2*^, Marzia Pucci^1*^, Marco Giallombardo^1^, Maria Antonietta Di Bella^1^, Mariacarmela Santarpia^3^, Pablo Reclusa,^4^* *Ignacio Gil-Bazo^5^**Christian Rolfo,^4#^ Riccardo Alessandro ^1,2#^*

**Supplementary Figures**

**
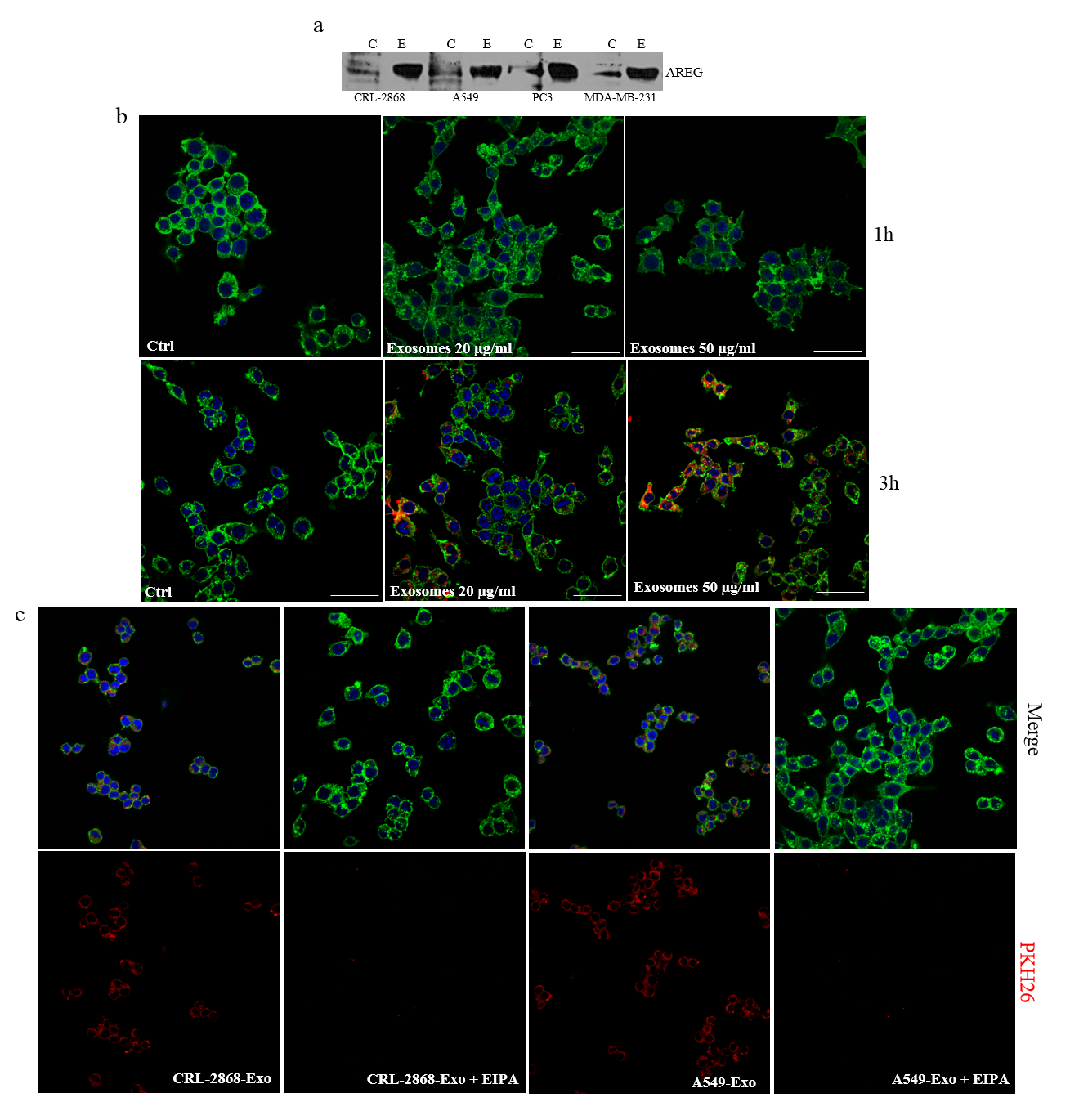
**

**Figure S1**


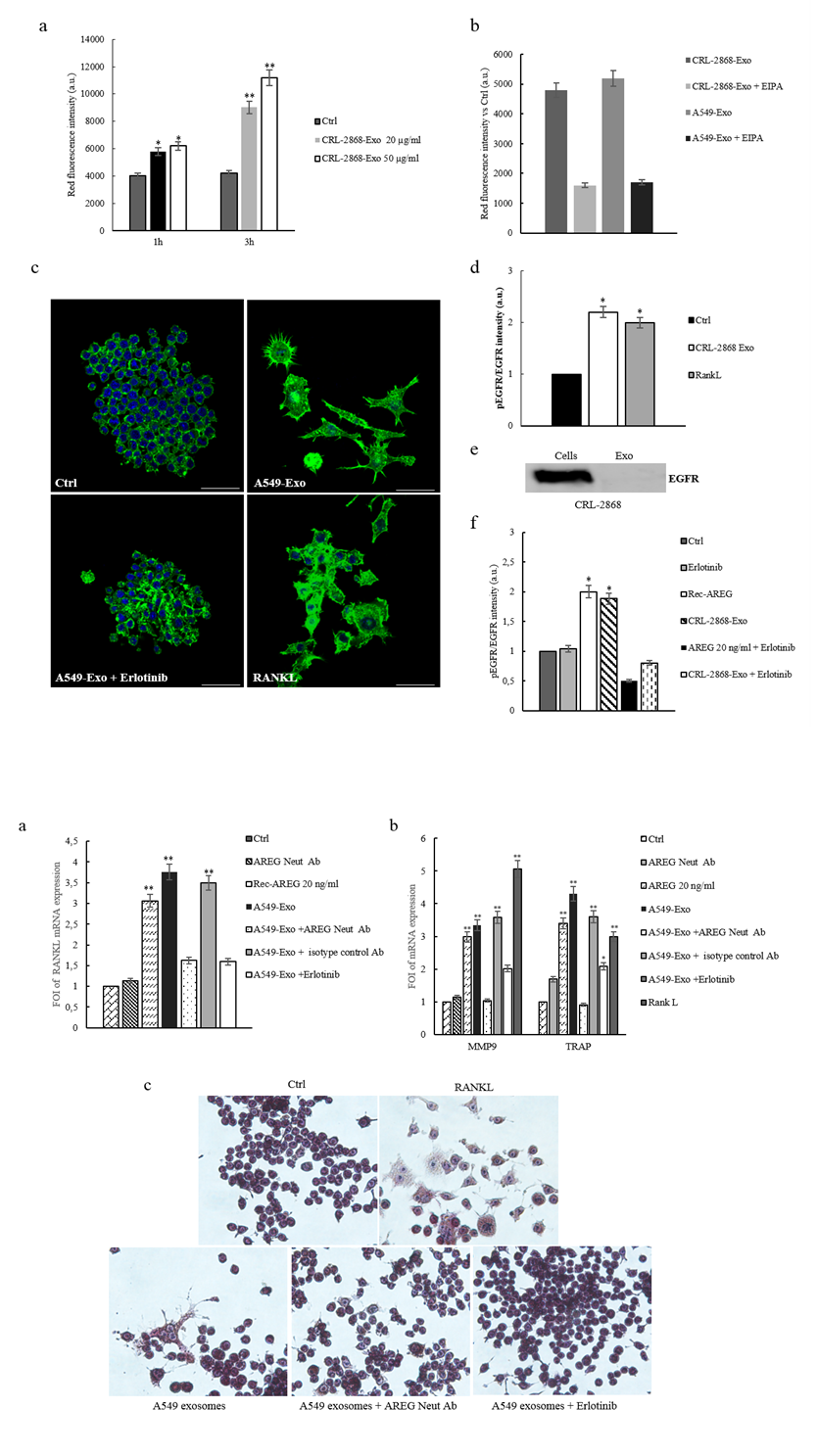


**Figure S2**

**Figure S3**


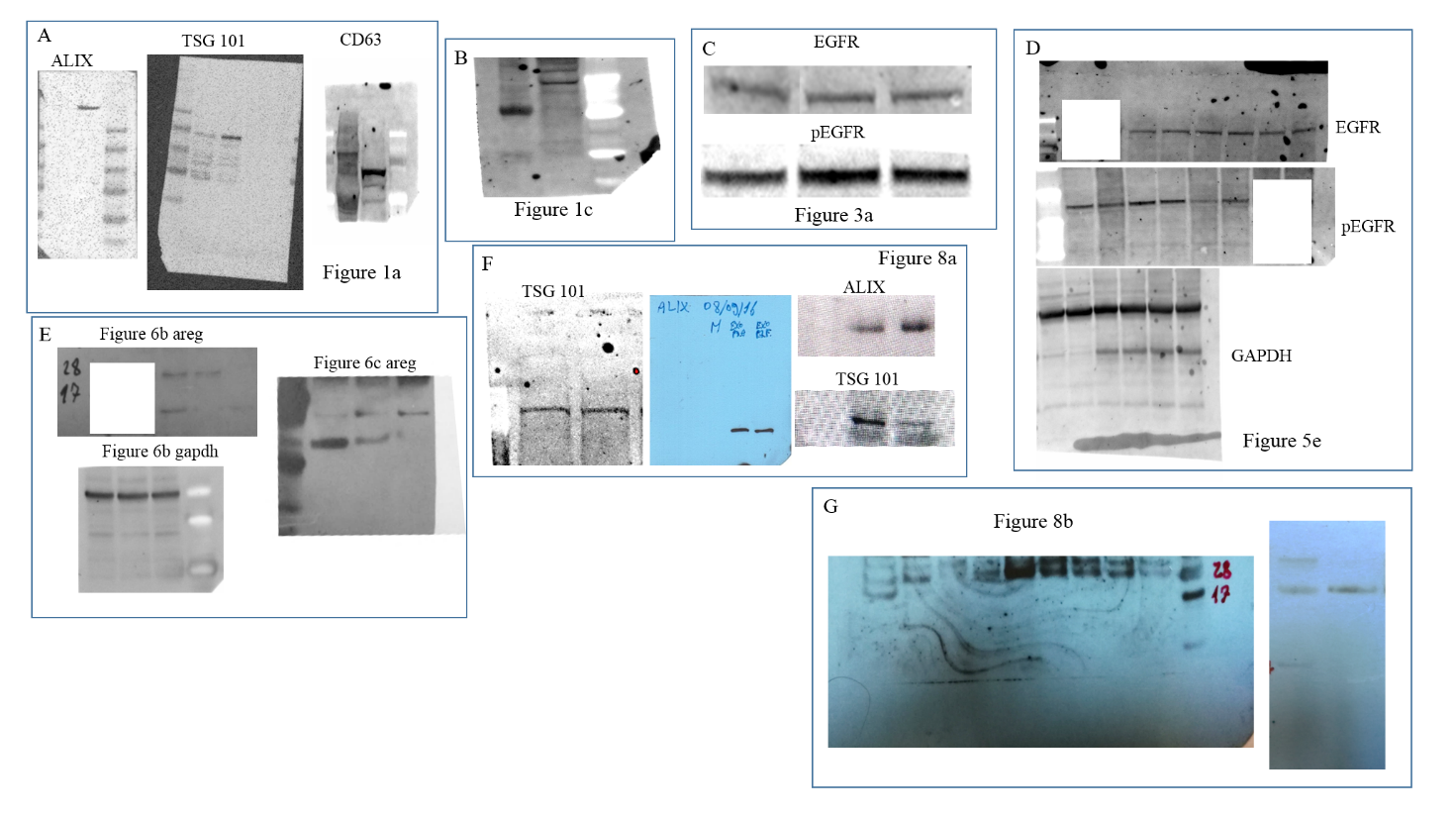

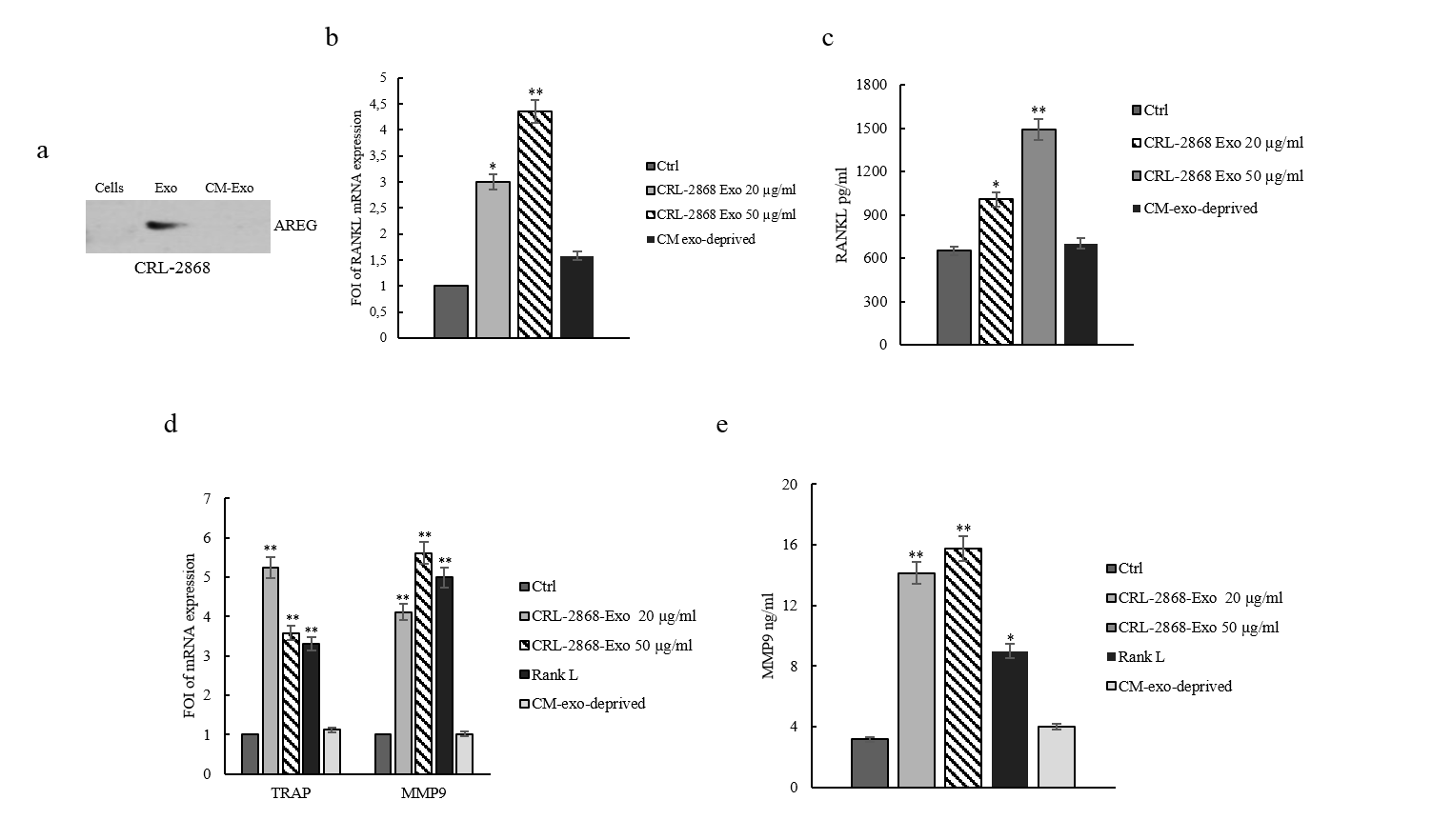


**Figure S4**

**Figure S5**

**Supplementary Methods**

**RNA extraction and real-time PCR**

RAW 264.7 cells were cultured in 12-well plates at 5.000 cells/ml for well and treated or not with CRL-2868-exosomes and A549 exosomes (20-50 µg/ml) or recombinant AREG (20-50 ng/ml) or RANK Ligand 25 ng/ml ± Erlotinib 0.5 µM for 6 days, as described in the results section. Neutralizing antibodies anti-AREG (Novus) (20 ng/ml) were incubated with CRL-2868 exosomes and A549 exosomes for 1h at 37°C and then used to treat RAW 264.7 cells for 6 days. CRL-2868 exosomes were incubated with non-specific antibodies for 1h at 37°C, as negative control.

Human primary preosteoclast cells were growth in 12-well plates at 1.5×10^6^ cells per well and treated or not with CRL-2868-exosomes (20-50 µg/ml) or recombinant AREG (20-50 ng/ml) or RANK Ligand 25 ng/ml ± Erlotinib 0.5 µM for 4 days, as described in the results. Human primary preosteoclast cells were, also treated with exosomes collected from plasma of patients NSCLC (20 µg/ml) for 4 days, as described in the results. RNA was extracted using the commercially available Illustra RNAspin Mini Isolation Kit (GE Healthcare, Little Chalfont, Buckinghamshire, UK), according to manufacturer’s instructions. Total RNA from RAW 264.7 or from human primary preosteoclast cells was reverse transcribed to cDNA using the High Capacity cDNA Reverse Transcription kit (Applied Biosystems, Foster City, CA, USA). RT-QPCR was performed in 48-well plates using the Step-One Real-Time PCR System (Applied Biosystems). For quantitative Sybergreen real-time PCR, reaction was carried out in a total volume of 20 µl containing 2 ˟ SYBER Green I Master Mix (Applied Biosystems), 2 µl cDNA and 300 nM forward and reverse primers. Primers are reported in Supplemental Table 1. All primers (Table 1) were obtained from Invitrogen (Foster City, CA, USA). Real-time PCR was performed in duplicates for each data point. Relative changes in gene expression between control and treated samples were determined using the ΔΔCt method. Levels of the target transcript were normalized to a GAPDH endogenous control, constantly expressed in all samples (∆Ct). For ∆∆Ct values, additional subtractions were performed between treated samples and control ∆Ct values. Final values were expressed as fold of induction.

**Western blotting and antibodies**

RAW 264.7 cells were cultured in petri dishes (p100) at 30.000 cells for each and treated or not with CRL-2868 exosomes (20-50 µg/ml) or recombinant AREG (20-50 ng/ml) or RANKL 25 ng/ml ± Erlotinib 0.5 µM for 6 days, as described in the results. Neutralizing antibodies anti-AREG (20 ng/ml) are incubated with CRL-2868-exosomes (50 µg/ml) for 1h at 37°C and then used to treat RAW 264.7 cells for 6 days. SDS-PAGE Electrophoresis and Western Blotting were performed as previously described (3). Briefly, cells were lysated for 1 hour and 30 minutes in lysis buffer containing 15mM Tris/HCl pH7.5, 120mM NaCl, 25mM KCl, 1mM EDTA, 0.5% Triton X100, and Protease Inhibitor Cocktail (100X, Sigma–Aldrich, USA). Cell lysates (from 30 μg to 50 µg per lane) were separated using 4-12% Novex Bis-Tris SDS-acrylamide gels (Invitrogen, Life Technologies, USA), transferred on Nitrocellulose membranes (Invitrogen, Life Technologies, USA), and immunoblotted with the primary antibodies. The following antibodies were used: EGFR, p-EGFR (Cell Signaling Technology, Lane Danvers, MA, USA), AREG (R&D Systems, Abingdon, UK), CD63 (sc-15363), were obtained from Santa Cruz Biotechnology (Santa Cruz Biotechnology, Inc., Santa Cruz, CA, USA); Alix (2171S) antibodies were obtained from Cell Signaling (Beverly, MA).

**ELISA assay**

RAW 264.7 conditioned medium (CM) was collected from cells stimulated or not for 6 days with CRL-2868-exosomes (20-50 μg/ml) or recombinant AREG (20-50 ng/ml) or RANKL 25 ng/ml ± Erlotinib 0.5 µM. Neutralizing antibody anti-AREG (20 ng/ml) was incubated with CRL-2868 cell-derived exosomes (50 µg/ml) for 1h at 37°C and used to treat RAW 264.7 cells for 6 days. Human primary osteoclasts conditioned medium (CM) was collected from cells stimulated for 4 days with OC medium alone or with CRL-2868-exosomes (20-50 µg/ml) ± Erlotinib 0.5 µM or with plasma patients (NSCLC) derived exosomes (20 µg/ml). CM aliquots were centrifuged to remove cellular debris and used to quantify MMP9 and RANK Ligand with ELISA kits according to the manufacturer’s protocol. MMP9 levels secreted by both Human primary OCs and Raw264.7 cells were quantified respectively by Human MMP-9 ELISA assays (Invitrogen) and mouse ELISA Kit for MMP9 Cloud-clone Corp®. RANK Ligand levels secreted by both Human primary OCs and RAW 264.7 cells were quantified respectively by ELISA Complete kit human sRANKL assay (KOMABIOTECH) and ELISA Complete kit mouse sRANKL assay (KOMABIOTECH). For details, see supplemental data.

**Supplementary Figure legends**

**Figure S1: a:** Detection by western blotting of AREG in 30 µg of CRL-2868, A549, PC3 and MDA-MB-231 exosomes compared to 30 µg of parental cells whole lysate. b: Confocal microscopy analysis of RAW 264.7 cells treated, for 1 and 3 hours, with 20 μg/ml (Exosomes 20 μg/ml) and 50 μg/ml (Exosomes 50 μg/ml) of A549 exosomes, compared to untreated RAW 264.7 cells (Ctrl). RAW 264.7 were stained with ActinGreen (green), nuclear counterstaining was performed using Hoescht (blue); exosomes were labelled with PKH26 (red). c: Analysis at confocal microscopy of RAW 264.7 cells co- treated, for 3 hour, with 50 µg/ml of CRL-2868 (CRL-2868-Exo + EIPA) and A549 exosomes (A549-Exo + EIPA) plus EIPA (50 μM), compared with RAW 264.7 cells treated, for 3 hour, with 50 µg/ml of CRL-2868 (CRL-2868-Exo) and A549 exosomes (A549-Exo). Scale bar = 10 µm.

**Figure S2: a:** Semi-quantitative analysis of CRL-2868 exosomes internalization, measured as red fluorescence intensity in the cytoplasm of RAW 264.7 cells. **b:** Semi-quantitative analysis of CRL-2868 exosomes internalization, measured as red fluorescence intensity in the cytoplasm of RAW 264.7 cells, after treatment with EIPA.

**c:** Confocal microscopy analysis of RAW 264.7 cells treated, for 6 days with: A549 exosomes, Erlotinib and A549 exosomes plus Erlotinib (A549-Exo + Erlotinib) and RANKL compared with RAW 264.7 control (Ctrl). Scale bar 10 µm. **d:** Densitometric analysis of Western blotting for pEGFR/EGRF showed in figure 3a. **e:** Western blot analysis of EGFR in 30 µg of CRL-2868 exosomes compared to 30 µg whole lysate of parental cells. **f:** Densitometric analysis of Western blotting for pEGFR/EGRF showed in figure 3a.

**Figure S3: a:** Evaluation by real Time PCR analysis of mRNA expression of RANKL in RAW 264.7 cells treated, for 6 days, with: AREG neutralizing antibodies, Rec-AREG (20 ng/ml), A549 exosomes, A549 exosomes plus AREG neutralizing antibodies, A549 exosomes plus isotype control antibodies and A549 exosomes plus Erlotinib. **b:** Evaluation by real Time PCR analysis of mRNA expression of TRAP and MMP9 in RAW 264.7 cells treated, for 6 days, with: AREG neutralizing antibodies, Rec-AREG (20 ng/ml), A549 exosomes, A549 exosomes plus AREG neutralizing antibodies, A549 exosomes plus isotype control antibodies, A549 exosomes plus Erlotinib and RANKL. Values are the mean ± SD of 3 three independent experiments *p≤ 0.05, **p≤ 0.01. **c:** TRAP staining of RAW 264.7 cells incubated with: A549 exosomes, RANKL, A549 exosomes plus AREG neutralizing antibodies, A549 exosomes plus Erlotinib, for 6 days, stained for TRAP and compared with untreated cells (Ctrl). Scale bar 10 µm.

**Figure S4: a:** Detection by western blotting of AREG in 30 µg of CRL-2868 exosomes (Exo) compared to 30 µg of parental cells whole lysate (Cells) and exosomes-deprived conditioned medium (CM-exo: 10 ml of medium concentrated from which 30 µg of CRL-2868 exosomes were isolated). **b:** Evaluation by quantitative Real Time PCR of mRNA RANKL expression in RAW 264.7 cells treated, for 6 days, with 20 and 50 μg/ml CRL-2868 exosomes and exosomes-deprived conditioned medium (CM-exo). **c:** msRANKL protein levels assessed by ELISA, in RAW 264.7 cells treated, for 6 days, with 20 and 50 μg/ml CRL-2868 exosomes and conditioned medium exosomes-deprived (CM-exo). **d:** Evaluation by quantitative Real Time PCR of mRNA expression of TRAP and MMP9 in RAW 264.7 cells treated, for 6 days, with 20-50 μg/ml CRL-2868 exosomes, RANKL (positive control) and exosomes-deprived conditioned medium (CM-exo). **e:** MMP9 protein level assessed by ELISA, in RAW 264.7 cells treated, for 6 days, with 20-50 μg/ml CRL-2868 exosomes, RANKL exosomes-deprived conditioned medium (CM-exo). Values are the mean ± SD of 3 three independent experiments *p≤ 0.05, **p≤ 0.01.

**Figure S5:** Original uncropped WBs of images reported in: **A:** Figure 1a; **B:** Figure 1b; **C:** Figure 3a; **D:** Figure 5e (The white shadow covered two samples out of this study); **E:** Figure 6b (The white shadow covered two samples out of this study) and 6c; **F:** Figure 8a; **G:** Figure 8b.

**TABLE 1**

| **Primer sequences** |  |  |
| --- | --- | --- |
| **Gene** | **Forward primer sequence (5'-3')** | **Reverse primer sequence (5'-3')** |
| mouse GAPDH | CCCAGAAGACTGTGGATGG | CAGATTGGGGGTAGGAACAC |
| mouse TRAP | GCGACCATTGTTAGCCACATACG | CGTTGATGTCGCACAGAGGGAT |
| mouse MMP9 | GCTGACTACGATAAGGACGGCA | GCGGCCCTCAAAGATGAACGG |
| mouse RANKL | TCTCTGGGTCTAACCCCTGG | CAGGTCCCAGCGCAATGTAA |
| human GAPDH | ATGGGGAAGGTGAAGGTCG | GGGTCATTGATGGCAACAATATC |
| human TRAP | GATCCTGGGTGCAGACTTCA | GCGCTTGGAGATCTTTAGAGT |
| human MMP9 | CGCTACCACCTCGAACTTTG | GCCATTCACGTCGTCCTTAT |
| human RANKL | AAGGCAGAATGTGTACCAGGG | CTTGCCTCTGGCTGGAAACC |
| human AREG | GTGGTGCTGTCGCTCTTGATACTC | TCAAATCCATCAGCACTGTGGTC |
